# Collaborative care for depression management in primary care: A randomized roll-out trial using a type 2 hybrid effectiveness-implementation design

**DOI:** 10.1016/j.conctc.2021.100823

**Published:** 2021-07-26

**Authors:** Justin D. Smith, Emily Fu, Jeffrey Rado, Lisa J. Rosenthal, Allison J. Carroll, Jacob A. Atlas, Andrew D. Carlo, Inger Burnett-Zeigler, Neil Jordan, C. Hendricks Brown, John Csernansky

**Affiliations:** a Department of Population Health Sciences, University of Utah School of Medicine, Salt Lake City, UT, USA; b Department of Psychiatry and Behavioral Sciences, Northwestern University Feinberg School of Medicine, Chicago, IL, USA; c Northwestern Medicine, Chicago, IL, USA; d Center of Innovation for Complex Chronic Healthcare, Hines VA Hospital, Hines, IL, USA

**Keywords:** Collaborative care, Depression, Hybrid design, Implementation, Mental health, Primary care, Study protocol

## Abstract

**Background:**

The Collaborative Care Model (CoCM) is a well-established treatment for depression in primary care settings. The critical drivers and specific strategies for improving implementation and sustainment are largely unknown. Rigorous pragmatic research is needed to understand CoCM implementation processes and outcomes.

**Methods:**

This study is a hybrid Type 2 randomized roll-out effectiveness-implementation trial of CoCM in 11 primary care practices affiliated with an academic medical center. The Collaborative Behavioral Health Program (CBHP) was developed as a means of improving access to effective mental health services for depression. Implementation strategies are provided to all practices. Using a sequential mixed methods approach, we will assess key stakeholders’ perspectives on barriers and facilitators of implementation and sustainability of CBHP. The speed and quantity of implementation activities completed over a 30-month period for each practice will be assessed. Economic analyses will be conducted to determine the budget impact and cost offset of CBHP in the healthcare system. We hypothesize that CBHP will be effective in reducing depressive symptoms and spillover effects on chronic health conditions. We will also examine differential outcomes among racial/ethnic minority patients.

**Discussion:**

This study will elucidate critical drivers of successful CoCM implementation. It will be among the first to conduct economic analyses on a fee-for-service model utilizing billing codes for CoCM. Data may inform ways to improve implementation efficiency with an optimization approach to successive practices due to the roll-out design. Changes to the protocol and current status of the study are discussed.

## Trial registration

ClinicalTrials.gov: NCT04321876. Registered: March 25, 2020. Retrospectively registered.

## 1 Background

Approximately 20 % of U.S. adults experience major depressive disorder in their lifetime, with an annual prevalence rate of 7–8% [1,2]. Depression is associated with significant burden and disability [3,4]. Numerous barriers prevent access to appropriate mental health care, including: national shortage of psychiatrists [5], especially in disadvantaged communities [6]; limited mental health insurance coverage despite parity legislation [7]; and stigma [8]. Thus, there is a need for more accessible evidence-based treatment opportunities.

Increasing numbers of patients present to their primary care provider (PCP) with mental health complaints. PCPs are the leading prescribers of psychotropic medications (e.g., antidepressants, anxiolytics, stimulants) [9,10], but may not have the resources or time to adequately treat mental health conditions. Evidence suggests that depressive disorders are underdiagnosed and undertreated by PCPs [11,12]. PCPs report difficulty connecting patients to mental health services [13], and only 30 %–50 % of patients follow through with external mental health referrals [14]. These factors highlight the ongoing need for accessible and effective primary care-based mental health treatment.

### 1.1 Collaborative Care Model (CoCM) for depression

CoCM is a model of mental health services delivered in primary care through systematic collaboration between PCPs, behavioral care managers (BCMs), and consulting psychiatrists [15,16]. The Improving Mood–Promoting Access to Collaborative Care Treatment (IMPACT) trial demonstrated that patients in CoCM, compared to usual care, were more likely to continue antidepressant treatment, achieve prolonged remission of depression, report greater quality of life and self-efficacy, and have lower risk of developing cardiovascular disease [[17], [18], [19], [20]]. Further, CoCM appears to be one of the best models for enhancing engagement in depression treatment among underserved racial/ethnic minority populations [21] and has been shown to provide good economic value [22,23].

### 1.2 Implementation of CoCM

Despite abundant trials demonstrating CoCM's clinical effectiveness, little research has sought to understand implementation [[24], [25], [26]]. Some guidance is provided by the University of Washington's AIMS Center [27,28] and others [29]. However, this guidance focuses largely on the intervention processes of CoCM and reimbursement models. Various studies have identified barriers, facilitators, and factors associated with high patient activation [[30], [31], [32], [33], [34], [35], [36]], including unclear referral processes, misunderstanding of CoCM, and reimbursement challenges. Frequently reported facilitators were extensive training in CoCM, supervision of BCMs, routine feedback/updates, and straightforward reimbursement systems. Anticipating such barriers and facilitators to CoCM implementation shaped the approach and evaluation used in the current study.

### 1.3 Study aims

The current study will examine the effectiveness and implementation of the Collaborative Behavioral Health Program (CBHP). As a Type 2 effectiveness-implementation trial, the co-primary aims are: 1) to evaluate the impact of our implementation strategy package on the progressive improvement in speed and quantity of CBHP implementation over successive practices; 2) to examine the acceptability and sustainability of CBHP, assessed via key stakeholder surveys, interviews, and focus groups, as well as rates of CBHP referral, engagement, and graduation; and 3) to test the effectiveness of CBHP for improving depression symptoms and spillover to common cardiovascular and cardio-metabolic syndromes.

## 2 Methods

### 2.1 Study design overview

This study will use a randomized roll-out implementation trial design [37,38] in 11 primary care practices affiliated with Northwestern Medicine, an academic medical center in Chicago, Illinois. All practices are randomized to the time at which CoCM implementation will begin. Sequential crossover of practices from control to intervention occurs in the roll-out schedule until all practices implement CoCM. Measurement of outcomes before and after the introduction of the intervention allows for within-practice comparisons, and replication across multiple practices affords between-practice comparisons that control for time and external factors. Roll-out implementation designs ensure confidence in the results of the evaluation because known and unknown biases are equally distributed in the case and control conditions [39]. Roll-out implementation designs are practical because many organizations feel it is unethical to withhold effective interventions, and these designs reduce the logistic and resource demands of delivering the strategy to all units simultaneously. The roll-out design of CBHP will allow for adaptation of the implementation plan in subsequent practices based on feedback from relevant stakeholders (e.g., PCPs, BCMs, healthcare system Operations department, and support staff).

### 2.2 Implementation and research team

For the current study, the members of the healthcare system involved in CBHP implementation will include PCPs (general internists, family medicine, advanced practice providers), support staff (nurses, medical assistants, other clinic staff), leadership (practice manager, Northwestern Medicine Operations department, Department of Psychiatry administration, chief medical officer, etc.), the BCM (social worker), and the consulting psychiatrist. In addition, one PCP per practice will be designated as the “PCP Champion” (PCP-C) either by volunteering for this role or being appointed by the practice manager. The PCP-C will serve as the point of contact for the research team, including completing intermittent research activities (i.e., surveys, qualitative interviews; see below). The primary members of the research team include two implementation scientists with expertise in mental health and behavioral interventions in primary care; four psychiatrists, three of whom are trained in and practice CoCM; two research-focused clinical psychologists with expertise in depression; a clinical psychology doctoral student; a health economist and mental health services researcher; and two members of the Northwestern Medicine Operations team housed in the Department of Psychiatry that lead the administrative responsibilities of CoCM for the healthcare system.

### 2.3 Participants, recruitment, eligibility, and practice-level randomization

**Fig. 1:**
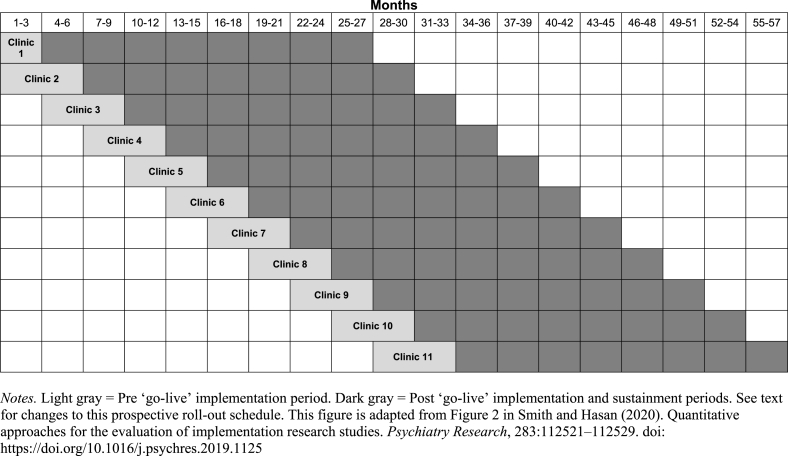
*Prospective randomized roll-out schedule and study periods*. *Notes.* Light gray = Pre ‘go-live’ implementation period. Dark gray = Post ‘go-live’ implementation and sustainment periods. See text for changes to this prospective roll-out schedule. This figure is adapted from Figure 2 in Smith and Hasan (2020). Quantitative approaches for the evaluation of implementation research studies. *Psychiatry Research*, 283:112,521–112529. doi: https://doi.org/10.1016/j.psychres.2019.1125

**Practices**. All 11 primary care practices associated with the academic medical center will be included. Matched-pair randomization will be used in the roll-out design to assign the sequence of CBHP implementation, as shown in Fig. 1. Two practices were identified as priorities for CBHP initiation by Northwestern Medicine Operations. A pseudorandom number generator was used to assign these two practices to the first and second roll-out positions, and the remaining nine practices were balanced and matched using the following practice-level variables from the prior fiscal year (2018): number of physicians, number of annual visits, number of patients, gender (% female), race (% white), proportion of Medicare recipients, and prior availability of co-located mental health services. Ultimately, 10,000 permutations were constructed in the possible ordering of the other nine practices by forming four pairs and one unmatched practice. Any design that provided the closest balance of the four practice pairs (and one unmatched practice) using Hotelling's T^2^ criterion would be considered to be nearly optimal, as this would lead to similar practices being implemented 3–6 months apart, thus allowing us to more accurately assess the process of early implementation. Rather than take the very best among these permutations, we randomly selected one assignment among those in the top 2 percentile so the order could not be deduced.

**Patients**. Once CBHP is implemented in the practice, primary care patients will be screened for elevated depressive symptoms using the Patient Health Questionnaire (PHQ-2 and PHQ-9; 47, 48) either during their regular primary care visits or after referral to CBHP by the BCM. When a PHQ-2 is elevated, PCPs receive a best practice alert to screen using the PHQ-9. Patients who endorse a score ≥10 on the PHQ-9 are eligible to be referred to CBHP. Patients are not eligible for CBHP if they require a higher level of care, including those with current suicidality, bipolar disorder, psychosis, or a primary diagnosis of substance use disorder or other severe mental health condition. BCMs will receive training to assist PCPs to make appropriate external referrals for these patients.

### 2.4 Intervention

This study will implement CBHP as a means of treating depression in primary care practices. CoCM has been widely implemented and described elsewhere [40]. We adhere to the University of Washington's AIMS Center version of CoCM. Critical components of CoCM are: a patient-centered care team comprising tripartite collaboration between PCPs, behavioral care managers (BCMs), and consulting psychiatrists; EHR or warm hand-off referral and individualized care planning; population-based care (EHR-based patient registry, caseload-focused consultation); a measurement-based, treat-to-target approach using biweekly depression symptom assessment in an outcome performance dashboard to achieve a score of <10 on the PHQ-9 and 50 % reduction in symptoms; evidence-based care for pharmacotherapy and psychotherapy (Behavioral Activation [41], Cognitive Behavioral Therapy [42], Motivational Interviewing [43]) for depression; and accountable care focused on achieving clinical outcomes (rather than increased volume of visits) and sustainable benefits for patients.

**Staffing**. Each practice will have a licensed clinical social worker who will perform the responsibilities of the BCM, with supplemental support for other mental health service needs (e.g., referral sources for serious mental illness or substance use disorders). BCMs may be assigned to one or two practices, depending on patient volume and flow. A consulting psychiatrist will be assigned to each practice to supervise and consult with the BCM. The primary care team, including the PCPs and support staff (e.g., nurses, medical assistants, administrative staff) in the practices will be trained in their respective roles in CoCM by an expert in CoCM from the research team. In alignment with prior research on barriers and facilitators to CoCM implementation, we viewed it as essential that the BCMs have an integrated role in the primary care team. This will be achieved via implementation strategies that include training and materials for all PCPs and practice support staff regarding the BCM's role in the team; having the BCM attend relevant practice meetings; designating space for the BCM in each practice; and promoting the BCM to serve as a mental health resource for the entire practice.

**Delivery of CoCM in Northwestern Medicine**. Patients with an elevated PHQ-9 (≥10) will be eligible to be referred to the BCM via a referral code in the EHR and, whenever possible, via a warm hand-off in the practice for immediate scheduling or completion of an in-person assessment. If the warm hand-off does not occur, the BCM will contact the patient within 1 week to schedule an in-person assessment. After completing the assessment, the BCM will present the case to the consulting psychiatrist and a treatment plan will be developed, which the BCM will communicate back to the PCP. CoCM specifies the BCM will follow-up with the patient weekly (completing at least two sessions per month), including at a minimum biweekly assessment of depressive symptoms using the PHQ-9. Sessions are typically conducted by telephone, though in-person sessions are available. All CBHP sessions are documented in the EHR and processed through the patient's insurance company [44]. The BCM also serves as a resource to the PCPs (e.g., external therapy referrals, substance use treatment options) and can provide psychotherapy for a small number of patients in CBHP. The consulting psychiatrist is also available to meet with the patients if clinically indicated. Consistent with the treat-to-target approach of CoCM, patients remain in the program until they achieve a reduction in symptoms as evidenced by two consecutive PHQ-9 scores <10 and a 50 % reduction in PHQ-9 scores measured over a 4-week period, at which point they are considered to have “graduated” from CBHP. Patients in CBHP who have undergone two adequate antidepressant trials and who do not achieve graduation will be referred to the psychiatry department at Northwestern Medicine for a higher level of care.

### 2.5 Procedures

**Implementation processes**. The process of CBHP implementation will be guided by the Exploration, Preparation, Implementation, and Sustainment (EPIS) model [45]. At our institution, implementation will be facilitated by several inherent factors as well as informed by previous research as described above. For example, this program generated top-down (e.g., appropriate financial remuneration for the BCM role in each practice) and bottom-up support (e.g., practice managers and the primary care steering committee supported the administration's decision to move forward with implementation) that will likely confer greater adoption and, ultimately, sustainment of CBHP. Northwestern Medicine Operations will lead the logistic preparation activities such as hiring of BCMs (none previously existed in the system), developing the health information technology infrastructures to support delivery (e.g., patient registry), and establishing EHR-based billing procedures using the CoCM billing codes [44].

***Depression screening***. The Northwestern Medicine Quality department will be responsible for the expansion of depression screening in primary care from only Medicaid-eligible patients age 65 years and older to all patients age 18 years and older at least once per year. A universal approach to screening was chosen to ensure a complete patient registry, to minimize missing patients who are eligible for CoCM, and to reduce bias of referring only patients with a depression diagnosis or medications in the EHR given aforementioned known problems with underdiagnosis and undertreatment of depression in primary care.

***Reimbursement for CoCM***. In contrast to much of the prior research on CoCM, a fee-for-service model will be employed. This economic model is made possible by two factors: negotiating with private and public payers (in 2018 and 2019) to cover CoCM and the recent enactment into law of Illinois bill SB2085 on January 1, 2020, which requires private insurers and Medicaid to cover the costs of CoCM. We have discussed our approach to CoCM billing under CPT codes 99,492–99494 elsewhere [44].

**Implementation Preparation period**. A 6-month period of implementation preparation will occur for each practice. During this time, Northwestern Medicine Operations initiates hiring of a BCM, the Department of Psychiatry is responsible for staffing the consulting psychiatrist role, The PCP-C is identified in the clinic, and the practice manager is enlisted in a number of activities (e.g., scheduling of training).

The newly hired BCM is asked to complete the online training modules from the American Psychiatric Association (https://www.psychiatry.org/psychiatrists/practice/professional-interests/integrated-care/get-trained) and shadow an experienced BCM in a Northwestern Medicine practice currently implementing CoCM for two weeks and receive on-site training from that practice's BCM for two days as CBHP is initiated in the new practice (the first practice in the roll-out shadowed a practice not included in the study that has been implementing CoCM since 2016). The PCP-C and consulting psychiatrist are also provided with the links to the online training modules specific to their roles and encouraged to complete them.

The PCP-C will begin to attend the monthly CBHP meeting, which is led by the implementation team's Operations lead. This meeting will include a number of people within the healthcare system that support CBHP implementation as well as members of the practices that are currently implementing CBHP and those that are within 6 months of their scheduled start date (are in implementation preparation). Finally, two meetings are scheduled at the practice: 1) One of the psychiatrist leads and the Department's Operations lead from the study team attend a physician or all-staff meeting to briefly introduce CBHP and alert the practice of the start date and schedule in-service trainings, and 2) the in-service trainings themselves.

***In-Service Trainings***. Approximately 1–2 months before CBHP implementation, a brief CBHP in-service training session will be conducted among the practice personnel during a regularly scheduled practice-wide staff meeting. Training sessions will be led by the practice's consulting psychiatrist, along with input from the BCM and Operations lead as appropriate. Training content will include an introduction to CBHP, an explanation of each team member's role, and procedures for screening, documentation, and billing. Due to the different roles and involvement in CBHP, the PCP and support staff trainings will be held separately for most practices. PCP trainings will additionally focus on the important role of CBHP in patient care and the mechanism by which PCPs can acquire support for behavioral health care more generally through CBHP. Support staff trainings will focus on their roles in screening patients. Team members who are unable to attend the training will be offered an informal training with the BCM or another member of the CBHP team at a later time. To evaluate fidelity, each training will be audio recorded and coded for degree of content coverage and duration of coverage for each component of the training.

**Implementation period**. The first day the BCM is available to see patients in a given practice is considered the start date of the CBHP implementation period in that practice (i.e., the “go-live date”). Implementation will last 12 months, followed by the Sustainment period (described below). All practices will have the same implementation supports; only the timing of implementation will vary by practice. The two primary implementation strategies that the team will employ as CBHP is rolled out are technical assistance and audit and feedback.

***Technical assistance***. Northwestern Medicine's Operations and Quality departments will assist with billing procedures, EHR modifications, the patient registry, depression screening, and ensuring each practice has a BCM. Northwestern's Department of Psychiatry and Behavioral Sciences will provide technical assistance regarding CoCM fidelity and related clinical issues arising in the delivery of CBHP. This assistance will be provided as requested by the practices and will also be offered when data indicate a need to address problems with referral and enrollment rates, engaging patients once enrolled in CBHP, and graduation rates of the program. These remediation and support services will be intertwined with the audit and feedback procedures.

***Audit and feedback procedures***. We will follow best practices for audit and feedback [46,47]. This process involves the feedback of program process and outcomes data to the practice manager, PCPs, BCM, consulting psychiatrist, and support staff, as indicated. At 90 days after the go-live date, a data report will be generated by the research team and presented to the PCPs to compare individual PCP referral rates (PCPs de-identified) with average practice-level referral rates as well as referral rates of other practices who are also implementing CBHP (not applicable to the first practice). Other variables, including time to assessment with the BCM, number of patients graduating CBHP, and other salient metrics will also be presented. These data will be presented every 90 days during practice staff meetings led by the practice manager and PCP-C, facilitated by the implementation team's Operations lead. A report will also be generated each quarter to be sent to Northwestern Medicine Operations and the co-chairs of the system's Primary Care Steering Committee.

**Sustainment period**. The sustainment period occurs between months 12 and 24 as per general consensus in the field of implementation science [48]. Support for implementation from the implementation team and Northwestern Medicine Operations will continue as previously described. The primary foci of the sustainment period concerns continued referral to CBHP, retention, and graduation rates for patients in the program, and ensuring the economic value to the healthcare system.

### 2.6 Implementation process, determinants, and outcomes measurement and analysis

The assessment procedures involve a sequential mixed methods approach [49] to (a) reduce stakeholder burden and increase odds of participation and response by Northwestern Medicine staff, and (b) obtain deeper information about the results seen on quantitative data sources through interviews and focus groups. Table 1 provides a summary of the measures, respondents/sources of data, and the assessment schedule for the project. All survey measures used in this study have been previously published and have demonstrated acceptable reliability and validity per the provided citations. There are two exceptions noted in the following sections that are provided as Additional File 1.

**Table 1:** Collaborative Behavioral Health Program measures, respondents, and assessment schedule.

Aims & Measures	CBHP TEAM MEMBERS							STUDY TIMELINE (months)								
	Study measures completed by:							Pre-Implementation	Go-Live	Implementation					Sustainment	
	EHR	PCPs	PCP-Champion	Practice Manager	Support Staff	BCM	Northwestern Medicine Operations	Post-Training (baseline)		3	4	6	12	15	18	24
Effectiveness Aims																
Patient-level	x											x	x	x	x	x
Clinic-level	x									x		x	x	x	x	x
Implementation Aims																
Interviews			x	x		X					x			x		x
Focus groups					x						x			x		
AAF-CoCM		x		x	x	X		X					x			x
CSAT			x	x									x			x
EBPAS		x		x	X	X		X					x			x
EBPQ		x		x	X	X		X					x			x
ILS		x		x	X	x		X					x			x
OCRBS		x		x	X	x		x					x			x
NoMAD			x	x		x							x			x
USIC (sources)			x			x	x									
Cost data (sources)	x					x	x									

*Notes*. PCPs include general internist, family medicine, and advanced practice providers. Support staff include nurses, medical assistants, and other clinic staff. USIC and cost data are continuously collected so only sources are listed here.

Abbreviations: BCM: behavioral care manager. EHR = electronic health record. PCP = primary care provider. OCRBS = Organizational Change Recipients' Beliefs Scale. ILS = Implementation Leadership Scale. EBPAS = Evidence-Based Practice Attitudes Scale. EBPQ = Evidence-Based Practice Questionnaire. AAF-CoCM = Acceptability, Appropriateness, Feasibility of Collaborative Care Model. USIC = Universal Stages of Implementation Completion.

**Pre-implementation baseline survey**. A pre-implementation survey will be distributed at or immediately following the CBHP in-service training via paper forms or an electronic link to the survey in Research Enterprise Data Capture [50]. Prior to completing the survey, all participants will complete a consent form. The survey will be tailored to each CBHP role: PCP/PCP-Cs (n = ~180), support staff (n = ~250), practice managers (n = ~11), and BCM (n = ~9). Comprising no more than 47 items (depending on role in CBHP), the survey includes a selection of validated implementation measures adapted for CBHP and tailored for each CBHP role following the same methods our group has used with previous survey administrations among diverse stakeholders [51]. A similar approach to Smith et al. [51] will be used for the CBHP study and validation of surveys using confirmatory factor analysis; invariance tests will also be performed.

Participants will first indicate their prior exposure to and experience with CBHP, CoCM, and similar programs. They will answer questions regarding the training they received specifically for CBHP (e.g., satisfaction, quality of the training materials, preparedness to implement). They will complete 8 items from the Organizational Change Recipients' Beliefs Scale (OCRBS) [52], 4 items from the Implementation Leadership Scale (ILS) [53], and 5 items from the Evidence-Based Practice Questionnaire (EBPQ) [54]. These shortened versions of established scales have been shown to be reliable and have good internal consistency in our team's prior work [55]. They will answer 10 items regarding the Acceptability, Appropriateness, Feasibility of Collaborative Care Model (AAF-CoCM), with questions developed for this study (see Additional File 1). Finally, participants will report their demographics (e.g., gender, age, race/ethnicity), bilingual abilities, and work history in the Northwestern Medicine system. Those who complete the survey will receive a $10 prepaid gift card.

**6-month follow-up survey**. The 6-month surveys are intended to examine the stakeholders’ perceptions of the CBHP implementation process. They will be distributed to PCPs and leadership via email or paper copies during a regularly scheduled practice meeting. The surveys include several of the same measures as the pre-implementation survey, including reviewing their experience with CBHP training, the OCRBS, the ILS, the EBPQ, and the AAF-CoCM. Participants will then be asked to rate the support they have received to implement CBHP from Northwestern Medicine and the implementation team, as well as completing open-ended questions to identify and comment on barriers and facilitators of CBHP at their practice. These questions were developed for this study (see Additional File 1). Those who complete the survey will receive a $10 prepaid gift card.

**12- and 24-month follow-up surveys**. The 12-month surveys were developed to examine the stakeholders’ perceptions of the process of CBHP implementation and plans for sustainment. They will be distributed to PCPs and leadership via email; these surveys will also be available in paper form during a regularly scheduled practice meeting. Similar to previous surveys, measures will include experience with CBHP training, the ILS, and the AAF-CoCM, as well as questions on CBHP support and satisfaction (see Additional File 1). PCPs will complete the 10-item Bergen Burnout Inventory [56] and the 23-item NoMAD tool [57] to assess PCP perspectives of the CBHP implementation process. The PCP-C and the practice manager additionally complete the Clinical Sustainability Assessment Tool (CSAT) Short Form [58,59], which assesses 7 domains (3 items each) related to sustaining evidence-based practices in clinical settings. The domains are: Engaged Leadership and Staff; Engaged Stakeholders; Planning and Implementation; Workflow Integration; Monitoring and Evaluation; Organizational Context and Capacity; Outcomes and Effectiveness. The CSAT has shown to be reliable, useable, and valid in a pilot study (n = 126) [58]. Scores (means, reliability statistics) on the CSAT will be compared to those in the literature for the Clinical version and its predecessor, the Program Sustainability Assessment Tool [60]. Those who complete the survey will receive a $10 prepaid gift card.

**Qualitative stakeholder interviews**. Four months (early in the implementation period), 15 months (end of implementation period), and 24 months (end of the sustainment period) after the go-live date, semi-structured qualitative stakeholder interviews will be individually conducted by members of the implementation team with the PCP-C, BCM, and practice manager. The 30-min interviews include questions about personal experiences with CBHP implementation, obstacles and problem-solving that arose since initiation of CBHP, and recommendations for sustaining CBHP in their practice, as well as the implementation of CBHP in other practices in the future (e.g., “*How have other professionals (PCPs/MAs/PAs/nurses/*support *staff) responded to CBHP?”*; “*For practices that do not have CBHP, how do you think they should go about launching or implementing the program? What specific things have worked well in your practice that they should replicate? What should they do differently*?”. The 4-month interview will additionally focus on how CBHP implementation is going so far, how collaborative relationships have developed, and what could be improved. The 15- and 24-month interviews will additionally focus on stakeholders’ perceptions of the relationships between primary care and the Department of Psychiatry and Behavioral Sciences, any additional behavioral health concerns or resources they would like to see addressed, and their opinions regarding the research aspects of CBHP (e.g., burden, relevance of questions). Interview participants will receive a $10 prepaid gift card.

Focus groups will be conducted with 5–8 support staff members using semi-structured interviews at the 4-, 15-, and 24-month time periods. Light food and beverages will be provided for focus group participants. Focus groups aim to understand the support staff's attitudes about CBHP as well as challenges and facilitators to screening all patients for depression (e.g., “*Concerning screening for depression, what has been your experience with the process and emphasis placed on screening all adults in the practice?”*).

All interviews and focus groups will be recorded, transcribed, and de-identified. Two to three transcripts will be randomly selected to identify themes to develop a codebook that will be used to code the remaining interviews. As roll-out proceeds, comments and themes from stakeholder interviews will be used to adapt protocols for future iterations of CBHP implementation. In order to integrate feedback in a timely manner, we use a quick and comprehensive qualitative analysis strategy called Framework-Guided Rapid Analysis [61] in which a structured template based on the Consolidated Framework for Implementation Research (CFIR) [62] analysis template will be generated. Interview question development was also informed by CFIR [62] to ensure coverage of relevant determinant domains. To further reduce the necessary time for qualitative data processing, we will provide ‘summary templates’ rather than the typically lengthy results from intensive coding. Implementation researchers have used this method successfully when quick turn-around is required [63]. All qualitative data processing and analysis will be done by a trained predoctoral clinical psychology trainee (EF) and faculty member on the research team (AJC), overseen by the PI (JDS). Disagreements in coding will be resolved via expert consensus among the investigative team. The mixed methods analytic approach will be a “merge the data” approach [49], which involves combining quantitative and qualitative data through complementarity [64,65]. We will also use our qualitative data to examine disparities on the quantitative implementation outcomes [66] described below.

**Practice-level implementation outcomes**. Measures of practice-level outcomes will include rates of depression screening, referral to CBHP, enrollment in CBHP, fidelity to CBHP using data available in the EHR and CBHP registry (i.e., enrollment of eligible patients, contact minimum two times monthly, PHQ-9 assessment biweekly), and speed and quantity of implementation using data from the Universal Stages of Implementation Completion® (USIC; [67]). EHR data will be the primary source to assess CoCM implementation. The RE-AIM evaluation framework extension for sustainability and equity [68] will be used for this study given our stated aims, part of which was previously described in Smith and Hasan [69]. Reach of CoCM (i.e., the proportion of patients seen in the practice in a given time period who are eligible for and referred to CoCM) will be compared by time from start-date and between practices. Reach rates will be calculated using a 3-month interval to most closely approximate potential users at any given point [70]. Additionally, the overall reach rate of the CBHP study will be compared to studies of CoCM implementation in the literature. Adoption of CoCM (i.e., the number of PCPs with eligible patients who provide CoCM referrals) will be calculated by tracking patient screening results and intakes completed by the BCM within the primary care clinician's encounter record. Both the total unique number of PCPs as well as the overall proportion will be calculated. Additionally, we will examine the trajectory of referrals by PCP over time by examining referral rates in 3-month intervals. Growth mixture models [71] will be used to test for the presence of unique groups of PCP referral trajectories. Consistent with the RE-AIM extension [68], we will examine equity in implementation outcomes as it concerns patient race/ethnicity, age, and gender, particularly as it pertains to PCP decisions to refer, patient's electing to enroll in CoCM, and graduation rates (i.e., effectiveness) of the program.

The USIC will be used to capture the speed of CBHP implementation and quantity of implementation activities completed at each practice. A version of the USIC adapted for CoCM has been shown to accurately assess implementation effectiveness of the model in rural primary care clinics and also detected site variations in performance [72]. Specifically, the USIC captures dates of implementation for 8 stages: 1) Engagement; 2) Readiness planning; 3) Implementation planning; 4) Staff hiring and initial training; 5) Fidelity assessment and monitoring; 6) Services and consultation; 7) Program adherence; and 8) Competency. The USIC will be updated for each site every two weeks, with respondents entering the date at which activities were completed in the prior two-week period. It will be completed by CBHP leaders, including consulting psychiatrists and the Department of Psychiatry Operations lead based on each role's direct knowledge of specific activities. Using the completion date of each activity, we will analyze the time elapsed in each practice to complete the activities of each stage (Duration Score). Then, we will calculate the percentage of stages completed (Proportion Score). These scores can then be used in statistical analyses to understand the factors that contributed to timely stage completion, the number of stages that are important for successful program implementation by relating the USIC to other implementation outcomes (such as reach rate), and simply whether there was a degree of improvement in implementation efficiency and scale as the rollout took place. That is, determining whether more stages were completed more quickly by later sites compared to earlier ones in the rollout schedule due to the implementation team learning from challenges and successes of earlier sites and being able to provide better support.

***Power Calculation***. Power estimate are based on 1250 unique patients per month per clinic (there were n = 164,022 across all 11 clinicals in FY18), a positive PHQ-9 rate ranging from 12.5 to 15 % [73,74], and a rate of enrollment in CBHP of 50–60 %. Concerning the roll-out schedule, thus far the time between each practice has varied between 1 and 6 months (Intervals: 3, 4, 6, 1, 4) with a plan for ~2-month interval for remaining practices. We hypothesize that CBHP will generate an increase in the appreciation of depressive symptoms and treatment initiation as evidence in the reach rate. Second, we hypothesize that implementation of CBHP will improve over time as earlier experiences will help guide practices randomly assigned to CBHP later in the roll out. Thus, we have two primary implementation outcomes of this roll-out design.

The first set of hypotheses involves comparison of screening rates to detect clinical levels of depressive symptoms, as well as treatment initiation rates pre-CHBP and post-CBHP among those screened positive. We rely on published data from four studies of CoCM in general adult primary clinics [[75], [76], [77]] to obtain reasonable rates of screening before (50 %) and after (75 %) CBHP is introduced, rates of positive screens (20 % both before and after CBHP), and rates of initiating treatment given a positive screen (30 % before and 50 % after CBHP). We assume a large ICC of 0.10 to account for variation in these rates across practices, both pre- and post-CBHP. Our power calculations are based on comparing records of patients in each of the 11 clinics in the 15 months of prior to initiating CBHP and 15 months afterwards, with an average of 8900 patients in each of the 11 clinics. We use a traditional 0.05 two-sided Type I error rate. All of these rate comparisons for the first hypotheses have exceptionally high power due to the large numbers of patients and long duration of the study. For example, we have greater than 99.9 % power to detect a significant improvement in screening for depression rates from 0.50 to 0.75; the power is 99.4 % for comparing 0.50 to 0.53, i.e., only a 6 % increase. For comparing rates of initiating new treatment before and after CBHP among those who are screened positive, we also have substantial power. If we assume that the rate of screening before CBHP is 0.50 and the rate afterwards is only modestly increased to 0.53; the screen positive rates are both 0.20, and the rates of initiating treatment given a positive screen are 0.30 before CBHP and 0.40 afterwards, power is 99 %; and even a 10 % increase from 0.30 to 0.33 achieves 96 % power.

In comparing Reach in these clinics prior to and after CBHP is delivered, we will focus first on comparing identification of individuals who have significant depressive symptoms through screening, first with PHQ-2 followed, if positive, with a PHQ-9; and second on the rate of new treatments for depression in the clinic. To compare a reasonable base rate for PHQ-2 screening of 30 % prior to CBHP, and a proportion of 50 % that would follow-up with a PHQ-9 test that turns out to be positive. This results in an anticipated 6 % of adults who would be eligible for entry into CBHP if it were offered (i.e., the counterfactual condition). Once CBHP is introduced to a clinic, we project an expected CoCM referral success of 75 % of those eligible (PHQ-9 positivity). We also expect 80 % of the clinicians post-CBHP will make at least one referral (i.e., adopt CBHP).

The second outcome addresses whether the implementation measures of reach and a composite index of USIC's duration (speed) and proportion (quantity) scores for each clinic show improvement across time as this design is rolled out to new clinics. We hypothesize that because of knowledge gained throughout this study, there will be a general improvement both in the reach as well as the speed and quantity of implementation over time. We calculate the power to detect a meaningful change in the rate of treatment initiation and USIC across the 11 clinics who were randomized to the order they implemented CBHP. For examining the improvement in initiating treatment over the 11 sites, we assume that each site begins with 30 % of those screening positive entering treatment, and the first site increases to 40 % by the end of 15 months on CBHP, displaying a modest improvement. We also assumed the last site to receive CBHP would start at the same value of 30 % but by the end of 15 months its treatment initiation rate would be higher than 40 %, which would be a consequence of improvement in implementation. Sites in the middle improve linearly between these two endpoints. Thus, the changes in treatment initiation across all sites and all times are dependent on the treatment initiation rate of the last site at the last time point. We calculated the power to detect a significant improvement as a function of this last time point. With a modest ICC of 0.02, we have at least 80 % power to detect a change in these rates if the last rate is 0.52, which corresponds to a 30 % increase over the 0.4 at the last time point for the first site. For very large ICCs of 0.10, we would have 80 % power if the last rate is 0.66, a 65 % increase over the corresponding last rate for the first site.

Treating a composite index of the USIC as a continuous measure, we next examined how much change we could detect over the 11 sites if there was a linear improvement. For a two-sided 0.05 level test, 80 % power is achieved if the change over time changed the USIC measure by an effect size of 0.95. Power could be improved substantially if a site level covariate at baseline were predictive of the USIC score. In analyses we will adjust for baseline levels of screening and treatment initiation; if these are correlated strongly (Pearson's *r* = 0.70), then we will have sufficient power to detect an overall standardized effect size change of 0.48.

As this study is continuing during the COVID-19 pandemic, we anticipate that there will be a reduction in referrals during the height of this exposure. We will explore whether such a drop would be explained simply by a reduction in primary care visits or whether the rate of referral for individuals who have screened positive on PHQ-9 is reduced. The latter is unlikely given findings of up to a threefold increase in depressive symptoms resulting from measures to slow the spread of COVID-19 [78].

***Cost of implementation***. The cost-effectiveness of CoCM has already been established [23]. Thus, we will conduct two types of economic analysis from the perspective of an implementing health system [79]. Our first economic approach will be an implementation cost analysis to estimate the budget impact to the healthcare system to implement and deliver CBHP. Second, we will conduct a cost offset analysis incorporating reimbursement from payers to ensure that costs to the system are recouped in such a way that CBHP can be sustained over time under current models of compensated care. The cost offset analysis will include determining whether healthcare expenditure costs have been averted by providing CBHP and improving depressive symptoms. We hypothesize that effective management of depressive symptoms will be related to improved chronic disease management, which should reduce the number of urgent care visits, emergency department visits, and hospitalizations.

### 2.7 Patient-level clinical effectiveness outcomes measurement

The clinical effectiveness of CBHP will primarily be measured by the reduction of depressive symptoms among patients referred to CBHP, as defined by clinically meaningful change using the reliable change index [80] on PHQ-9 scores. Data are collected as part of routine patient evaluation and care in CBHP, rather than for the purpose of the research study. Specifically, de-identified patient data will be pulled from the EHR by the Northwestern University Enterprise Data Warehouse. Between-practice comparisons will be computed, as will comparisons by time within-practice and time of roll-out with the hypothesis that as CBHP implementation improves, patient outcomes will follow suit. Additionally, we will evaluate the impact of CBHP (i.e., improving depression symptom management) with co-occurring cardiovascular (e.g., heart failure, blood pressure, cholesterol), cardio-metabolic (e.g., diabetes, insulin resistance), and other chronic health conditions (e.g., obesity, asthma) identified through EHR query.

For patient-level clinical effectiveness outcomes, see Table 2 for the number of providers practicing and patients attending each of the 11 practices. Based on 97,931 unique patients with primary care visits during the 2018 fiscal year, the national 12-month depression prevalence of between 4 % [81] and 8 % [82], and accounting for ineligibility (e.g., severe mental illness) or refusal, we very conservatively estimate that at least 5 % of patients (n = 4700) will be eligible to be referred to CBHP in a given year once all practices have implemented CBHP.

**Table 2:** Primary care providers and unique patients with a primary care visit per enrolled practice (Fiscal Year 2018).

Primary care Practice	N providers	N patients
1	8	4177
2	33	12,192
3	4	2459
4	12	9571
5	17	5273
6	7	4310
7	12	9521
8	10	3525
9	42	31,321
10	8	3849
11	27	11,733
**TOTAL:**	**180**	**97,931**

We will conduct three analyses pertaining to effectiveness of CBHP in reducing depression: 1) rate of CBHP graduation (total % graduated from CBHP/eligible enrolled patients in CBHP controlling for total population effect using existing data from synthesis trials using repeat PHQ-9 scores and reductions without intervention); 2) between-site comparison of graduation rates; and 3) effect of time from go-live date on graduation rate (potential for improvement in program outcomes over time). Given that effectiveness of CBHP is well-established, we have not conducted a formal power analysis.

### 2.8 Understanding failures to link referred patients to CoCM

It is anticipated that ~20–25 % of patients who are eligible and are referred to CBHP will not enroll in the program or will be lost to follow-up. To understand reasons for this failure-to-link, a subset of participants (n = 80) who either declined the referral to CBHP or whom the BCM failed to reach after referral to the program (defined as called two times and left voice messages but no contact with patient after referral) will be identified through the EHR. They will then be contacted via a mailed letter, email, or telephone call inviting them to complete a 15-item survey delivered either verbally over the phone or electronically. At the end of the survey, approximately n = 40 participants will also be offered the opportunity to participate in a 6-question semi-structured interview, informed by patient-centered domains of CFIR [62], via telephone. If the participant does not respond within 10 business days of being contacted, the research team will attempt to contact the participant by telephone up to six times. Participants will be compensated via prepaid gift cards: $10 for completing the survey and $10 for participating in the semi-structured interview. The goals of examining patients that we failed-to-link to CBHP are to 1) understand how to modify or introduce new implementation strategies within the system, particularly for each of the two patient phenotypes (declined referral and failed-to-reach), and 2) explore issues of equity by examining group differences by patient race/ethnicity, age, and gender.

### 2.9 Data management

All survey data collection will be centralized in Northwestern University's REDCap system [50]. REDCap is a web-based, electronic data capture software solution and workflow methodology for designing clinical and translational research databases. The web application is hosted entirely on Northwestern University's IT servers and secured by the University's infrastructure and network security protocols. Surveys will be administered using paper forms or electronically through emailed survey invitation links via REDCap. Surveys administered on paper forms will be manually entered into REDCap, after which the paper forms will be appropriately destroyed. The study team will collect a minimal number of identifiers from survey respondents for purposes of follow-up survey tracking; however, these identifiers will be clearly marked in REDCap and will remain only on secure Northwestern University servers. The study data manager will restrict REDCap user rights and ensure that data export functionality is limited to de-identified exports.

## 3 Discussion

CoCM is a well-established program for treatment of depression in primary care [18] that has been shown to be highly acceptable to participating patients and with patient input regarding implementation [83,84]. The current study aims to assess the effectiveness and implementation of CoCM among 11 primary care practices in a health system that features a large academic medical center. There are several strengths of our approach to evaluating implementation and effectiveness in this trial. The roll-out implementation design, with matched-pair randomization assigning CoCM to begin in a new practice every 3–6 months, will afford the opportunity to optimize implementation with progressive iterations that may be assessed by statistical comparisons between paired practices. Rapid qualitative analysis of implementation challenges and drivers ensures timely, data-driven decisions for improving implementation planning and support for the both current and future practices. These data can also be used as part of the audit and feedback strategy to improve implementation within practices by reporting back to stakeholders (CBHP research team members, Operations/Administrative) and adjusting implementation supports accordingly.

This design is also highly aligned with the health system's processes for hiring new staff and otherwise allocating resources equitably as there is both an advantage to beginning early (mental health services are available to patients) and to beginning later in the roll-out (implementation of CBHP improves). Despite the corpus of research on the effectiveness of CoCM for depression in primary care, there remains a dearth of rigorous research focused on how best to support adoption and sustained implementation over time [83] and across multiple sites within the same health system. Given the vast evidence in the literature regarding the acceptability of CoCM to patients, we elected to focus on implementation by PCPs and the healthcare system. However, patient perspectives are critical to understanding implementation challenges and to identification of solutions. As our sub study of failure-to-link to CBHP exemplifies, we intend to involve patients in deeper understanding of implementation deficiencies that are identified in the data and the process of implementing CBHP. Additional opportunities to hear patients' voices and learn from their experiences will be identified over the course of the study.

This Type 2 hybrid effectiveness-implementation trial can serve as an opportunity to identify key drivers of CoCM and its downstream effects on patient depression outcomes. The implementation strategies involved in this study are highly compatible with typical procedures for implementing new practices and quality improvement initiatives in contemporary healthcare systems. Training, ongoing technical assistance, development of health information technologies (e.g., EHR tools, patient registry), and audit and feedback approaches are routinely used in Northwestern Medicine specifically. This study could indicate that more intensive implementation strategies, such as practice facilitation, might be needed for some or all practices to implement CoCM. Additional research on the effects of specific strategies will undoubtedly be needed. We also envision successful implementation of CBHP for depression laying the foundation for expanding the model to other common mental health conditions, such as anxiety, substance abuse, trauma, and cognitive problems (e.g., dementia). Although CoCM has been used for the treatment of anxiety [85], bipolar disorder [86], substance abuse [87], and other problems [88,89], these adaptations have thus far not been widely implemented as has been the case for depression.

### 3.1 Current status and changes to the protocol

At the time of this submission, we have started CBHP in all 11 practices. The earliest starting practices in the roll-out schedule have entered the sustainment phase and completed the 15-month interviews and focus group. A number of substantive changes to the protocol have occurred that are worth noting.

First, there have been a number of changes to the roll-out schedule. The order was switched for two of the practices when the practice slated to go next experienced the sudden departure of both the practice manager and a senior PCP who was involved in CBHP preparations. It was determined that it would not be advisable to attempt a large-scale practice change like CBHP without the new leadership in place. Thus, the order was simply switched with the next practice. This is considered a minor protocol violation as these were matched pairs and order will not affect the soundness of the randomization scheme. Additionally, based on increased demand for mental health services by the primary care practices, the healthcare system decided to accelerate the pace of roll-out from every 3–4 months to approximately every 2 months for the final 6 clinics. Accelerated roll-out will be made possible by having BCMs split across two practices while referrals ramp up during the early stages of CBHP, thus allowing time for the hiring of new BCMs as demands rise. Hiring of qualified BCMs was expected to be one of the key challenges for an accelerated roll-out.

Second, the planned audit and feedback procedures have been done in very limited fashion due to challenges in obtaining the necessary data-based metrics from the health system's Enterprise Data Warehouse. As a result, we have focused on depression screening rates and provider- and practice-level referral rates to CBHP to this point as these two metrics are vital to identifying and then potentially linking patients to CoCM. Efforts to obtain the needed data are underway and progressing well but the proposed audit and feedback procedures have largely not occurred as planned to this point.

Third, due to the need to increase treatment options for behavioral health beyond depression, the healthcare system expanded inclusion criteria for CBHP to include patients with anxiety (in the absence of depression). Referral and enrollment for anxiety only will be tracked and changes to the analytic plan will occur accordingly.

Fourth, the healthcare system decided to expand the implementation of CBHP beyond the 11 practices included in this study thus far. With the implementation of CBHP in these 11 practices, all the practices in Northwestern Medicine's Central Region will now provide CoCM. Thus far, the North Region is implementing CBHP in two of their six primary care practices with plans for a slower roll-out of one practice every 6 months. These practices will be included in the overall CBHP study and are following the same protocol as described here for the Central Region practices. The addition of these six practices in the North Region represent the start of a cumulative trial, that is, a trial where one plans to combine separately funded studies that share the same underlying interventions, implementation strategies, measurement, and design protocols [55]. Cumulative trials provide substantial increases in statistical power and are especially valuable in examining whether implementation and effectiveness outcomes improve over time as knowledge is gained [90]. Although no timeline is yet established, it is likely that Northwestern Medicine's West and Northwest Regions will also implement CoCM if the results of this study indicate that it is effective for patients, acceptable to stakeholders in the system, and is economically viable.

Finally, we write this manuscript in the midst of the COVID-19 pandemic which included a statewide shelter in place order in Illinois in Spring 2020. Northwestern Medicine, like most healthcare systems in the state, moved entirely to telemedicine services for an extended period of time for indicated primary care visits and reallocation of resources and personnel to address the COVID-19 response in the Chicago area. As a result, the planned roll-out and research evaluations were temporarily suspended in Spring 2020 until primary care practices settled into their new workflows. We have since resumed the roll-out as planned with the remaining clinics in the predetermined randomization order. The evaluations were adapted accordingly given that enrolled clinics have switched to virtual staff meetings and have varying depression screening procedures and support staff involvement. For example, qualitative interviews are conducted virtually, surveys are distributed electronically, and support staff focus groups are on-hold due to burden and limited CBHP involvement. This change to the protocol will undoubtedly impact new referrals to the program from those practices already implementing CBHP given disruptions to primary care. But the roll-out design will allow us to test this unanticipated disruption empirically using an interrupted time-series-type analysis to determine the impact [91]. Additionally, new questions about the impact of COVID-19 on CBHP and clinic workflow have been added to the 6, 12 and 24-month surveys and all qualitative interviews. This will aid us in understanding the effects of the COVID-19 response on CBHP implementation and program effectiveness. At present, the majority of primary care services can be resumed in person as vaccination rates increase, however, telemedicine is still being widely used for most primary care visits and CBHP contacts between patient and BCM and the BCM and PCPs. The overall analysis and aims of the study are unlikely to be significantly affected by this or any other of the protocol modifications.

## Ethics approval and consent to participate

This study was approved by Northwestern University's Institutional Review Board (STU00208338). Written informed consent was obtained electronically prior to survey administration, stakeholder interviews, and focus groups for practice staff. Northwestern Medicine's Electronic Data Warehouse consent procedures provide access to patient-level data without express consent for this study as those patients opting out of data sharing are removed from the dataset prior to delivery to the research team.

## Consent for publication

Not applicable.

## Availability of data and material

Data and materials are available upon request to the corresponding author.

## Funding

This work was supported by a grant from the Woman's Board of Northwestern Memorial Hospital, awarded to JC with JS serving as principal investigator. The funder reviewed the study protocol as part of the application process, but has no role in study design, data collection, analysis or publication. Additional support for this research project was provided by 10.13039/100000026National Institute on Drug Abuse grant P30DA027828 that supports the Center for Prevention Implementation Methodology for Drug Abuse and HIV, awarded to CB. JS also received support by the Implementation Research Institute (IRI) at the George Warren Brown School of Social Work, Washington University in St. Louis through an award from the 10.13039/100000025National Institute of Mental Health (5R25MH08091607) and the 10.13039/100000738Department of Veterans Affairs, Health Services Research & Development Service, 10.13039/100007181Quality Enhancement Research Initiative (10.13039/100007181QUERI). REDCap and the Northwestern Medicine Enterprise Data Warehouse (EDW) are supported by the 10.13039/100007059Northwestern University Clinical and Translational Science (NUCATS) Institute, Research reported in this publication was supported, in part, by the National Institutes of Health's 10.13039/100006108National Center for Advancing Translational Sciences, Grant Number UL1TR001422. The content is solely the responsibility of the authors and does not necessarily represent the official views of the National Institutes of Health or the Department of Health and Human Services.

## Authors’ contributions

JS, CB, and JC conceptualized and designed the study. JS, EF, and AC led the development of the manuscript. JS, EF, LR, JR, JA, CH, and IB-Z selected the surveys, adapted the survey items, and developed the interview and focus group questions. CB conducted the power analyses. All authors critically reviewed the manuscript and contributed text to their respective sections and areas of expertise on the project.

## Declaration of competing interest

The authors declare that they have no known competing financial interests or personal relationships that could have appeared to influence the work reported in this paper.

The authors declare the following financial interests/personal relationships which may be considered as potential competing interests:

Andrew D. Carlo, MD MPH works as a part-time consultant for Meadows Mental Health Policy Institute.

Andrew D. Carlo, MD MPH, Jacob A. Atlas, BS, Jeffrey Rado, MD MPH, John Csernansky, MD, Inger Burnett-Zielger, PhD, and Lisa Rosenthal, MD receive salary support from Northwestern Medicine.

## List of Abbreviations

AAF-CoCM: Acceptability, Appropriateness, Feasibility of Collaborative Care Model
BCM: Behavioral care manager
CBHP: Collaborative Behavioral Health Program
CoCM: Collaborative Care Model
EBPAS: Evidence-Based Practice Attitudes Scale
EBPQ: Evidence-Based Practice Questionnaire
EHR: Electronic health record
ILS: Implementation Leadership Scale
OCRBS: Organizational Change Recipients' Beliefs Scale
PCP: Primary care provider
PCP-C: PCP Champion
PHQ-9/2: Patient Health Questionnaire (9 and 2 item versions)
USIC: Universal Stages of Implementation Completion

## References

[1] National Institute of Mental Health (2019). Major Depression.

[2] Brody D.J., Pratt L.A., Hughes J.P. (2018). Prevalence of depression among adults aged 20 and over: United States, 2013-2016. NCHS Data Brief.

[3] Katon W., Lin E.H.B., Kroenke K. (2007). The association of depression and anxiety with medical symptom burden in patients with chronic medical illness. Gen. Hosp. Psychiatr..

[4] World Health Organization (2017). Depression and Other Common Mental Disorders: Global Health Estimates.

[5] Bishop T.F., Seirup J.K., Pincus H.A., Ross J.S. (2016). Population of US practicing psychiatrists declined, 2003-13, which may help explain poor access to mental health care. Health Aff..

[6] Cummings J.R., Allen L., Clennon J., Ji X., Druss B.G. (2017). Geographic access to specialty mental health care across high- and low-income US communities. JAMA Psychiatry.

[7] (2000). Mental Health Parity Act: Despite New Federal Standards, Mental Health Benefits Remain Limited.

[8] Corrigan P.W., Druss B.G., Perlick D.A. (2014). The impact of mental illness stigma on seeking and participating in mental health care. Psychol. Sci. Publ. Interest.

[9] Olfson M., Kroenke K., Wang S., Blanco C. (2014). Trends in office-based mental health care provided by psychiatrists and primary care physicians. J. Clin. Psychiatr..

[10] Mark T.L., Levit K.R., Buck J.A. (2009). Datapoints: psychotropic drug prescriptions by medical specialty. Psychiatr. Serv..

[11] Cepoiu M., McCusker J., Cole M.G., Sewitch M., Belzile E., Ciampi A. (2008). Recognition of depression by non-psychiatric physicians--a systematic literature review and meta-analysis. J. Gen. Intern. Med..

[12] Von Korff M., Shapiro S., Burke J.D., Teitlebaum M., Skinner E.A., German P. (1987). Anxiety and depression in a primary care clinic. Comparison of diagnostic interview schedule, general health questionnaire, and practitioner assessments. Arch. Gen. Psychiatr..

[13] Cunningham P.J. (2009). Beyond parity: primary care physicians' perspectives on access to mental health care. Health Aff..

[14] Fisher L., Ransom D.C. (1997). Developing a strategy for managing behavioral health care within the context of primary care. Arch. Fam. Med..

[15] Katon W., Von Korff M., Lin E., Walker E., Simon G.E., Bush T. (1995). Collaborative management to achieve treatment guidelines. Impact on depression in primary care. J. Am. Med. Assoc..

[16] Gunn J., Diggens J., Hegarty K., Blashki G. (2006). A systematic review of complex system interventions designed to increase recovery from depression in primary care. BMC Health Serv. Res..

[17] Unützer J., Katon W., Callahan C.M., Williams J.W., Hunkeler E., Harpole L. (2002). Collaborative care management of late-life depression in the primary care setting: a randomized controlled trial. J. Am. Med. Assoc..

[18] Hunkeler E.M., Katon W., Tang L., Williams J.W., Kroenke K., Lin E.H.B. (2006). Long term outcomes from the IMPACT randomised trial for depressed elderly patients in primary care. BMJ.

[19] Stewart J.C., Perkins A.J., Callahan C.M. (2014). Effect of collaborative care for depression on risk of cardiovascular events: data from the IMPACT randomized controlled trial. Psychosom. Med..

[20] Van Leeuwen Williams E., Unützer J., Lee S., Noël P.H. (2009). Collaborative depression care for the old-old: findings from the IMPACT trial. Am. J. Geriatr. Psychiatr..

[21] Interian A., Lewis-Fernández R., Dixon L.B. (2013). Improving treatment engagement of underserved U.S. racial-ethnic groups: a review of recent interventions. Psychiatr. Serv..

[22] Grochtdreis T., Brettschneider C., Wegener A., Watzke B., Riedel-Heller S., Härter M. (2015). Cost-effectiveness of collaborative care for the treatment of depressive disorders in primary care: a systematic review. PLoS One.

[23] Jacob V., Chattopadhyay S.K., Sipe T.A., Thota A.B., Byard G.J., Chapman D.P. (2012). Economics of collaborative care for management of depressive disorders: a community guide systematic review. Am. J. Prev. Med..

[24] Solberg L.I., Crain A.L., Maciosek M.V., Unützer J., Ohnsorg K.A., Beck A. (2015). A stepped-wedge evaluation of an initiative to spread the collaborative care model for depression in primary care. Ann. Fam. Med..

[25] Solberg L.I., Crain A.L., Jaeckels N., Ohnsorg K.A., Margolis K.L., Beck A. (2013). The DIAMOND initiative: implementing collaborative care for depression in 75 primary care clinics. Implement. Sci..

[26] Rossom R.C., Solberg L.I., Magnan S., Crain A.L., Beck A., Coleman K.J. (2017). Impact of A National collaborative care initiative for patients with depression and diabetes or cardiovascular disease. Focus.

[27] Katon W., Unützer J., Wells K., Jones L. (2010). Collaborative depression care: history, evolution and ways to enhance dissemination and sustainability. Gen. Hosp. Psychiatr..

[28] Bao Y., Druss B.G., Jung H.-Y., Chan Y.-F., Unützer J. (2015). Unpacking collaborative care for depression: examining two essential tasks for implementation. Psychiatr. Serv..

[29] Goodrich D.E., Kilbourne A.M., Nord K.M., Bauer M.S. (2013). Mental health collaborative care and its role in primary care settings. Curr. Psychiatr. Rep..

[30] de Jong F.J., van Steenbergen-Weijenburg K.M., Huijbregts K.M.L., Vlasveld M.C., Van Marwijk H.W.J., Beekman A.T.F. (2009). The Depression Initiative. Description of a collaborative care model for depression and of the factors influencing its implementation in the primary care setting in The Netherlands. Int. J. Integrated Care.

[31] Møller M.C.R., Mygind A., Bro F. (2018). Who needs collaborative care treatment? A qualitative study exploring attitudes towards and experiences with mental healthcare among general practitioners and care managers. BMC Fam. Pract..

[32] Curran G.M., Pyne J., Fortney J.C., Gifford A., Asch S.M., Rimland D. (2011). Development and implementation of collaborative care for depression in HIV clinics. AIDS Care.

[33] Whitebird R.R., Solberg L.I., Jaeckels N.A., Pietruszewski P.B., Hadzic S., Unützer J. (2014). Effective Implementation of collaborative care for depression: what is needed?. Am. J. Manag. Care.

[34] Svenningsson I., Petersson E.L., Udo C., Westman J., Björkelund C., Wallin L. (2019). Process evaluation of a cluster randomised intervention in Swedish primary care: using care managers in collaborative care to improve care quality for patients with depression. BMC Fam. Pract..

[35] Taylor A.K., Gilbody S., Bosanquet K., Overend K., Bailey D., Foster D. (2018). How should we implement collaborative care for older people with depression? A qualitative study using normalisation process theory within the CASPER plus trial. BMC Fam. Pract..

[36] Wozniak L., Soprovich A., Rees S., Al Sayah F., Majumdar S.R., Johnson J.A. (2015). Contextualizing the effectiveness of a collaborative care model for primary care patients with diabetes and depression (teamcare): a qualitative assessment using RE-AIM. Can. J. Diabetes.

[37] Hwang S., Birken S.A., Melvin C.L., Rohweder C.L., Smith J.D. (2020). Designs and methods for implementation research: advancing the mission of the CTSA program. Journal of Clinical and Translational Science.

[38] Brown C.H., Curran G., Palinkas L.A., Aarons G.A., Wells K.B., Jones L. (2017). An overview of research and evaluation designs for dissemination and implementation. Annu. Rev. Publ. Health.

[39] Grimshaw J., Campbell M., Eccles M., Steen N. (2000). Experimental and quasi-experimental designs for evaluating guideline implementation strategies. Fam. Pract..

[40] Archer J., Bower P., Gilbody S., Lovell K., Richards D., Gask L. (2012). Collaborative care for depression and anxiety problems. Cochrane Database Syst. Rev..

[41] Martell C.R., Dimidjian S., Herman-Dunn R. (2013). Behavioral Activation for Depression: A Clinician's Guide.

[42] Beck A.T., Rush A.J., Shaw B.R., Emery G. (1979). Cognitive Therapy of Depression.

[43] Miller W., Rollnick S. (2002). Motivational Interviewing: Preparing People for Change.

[44] Wolk C.B., Alter C.L., Kishton R., Rado J., Atlas J.A., Press M.J. (2021). Improving payment for collaborative mental health care in primary care. Med. Care.

[45] Moullin J.C., Dickson K.S., Stadnick N.A., Rabin B., Aarons G.A. (2019). Systematic review of the exploration, preparation, implementation, sustainment (EPIS) framework. Implement. Sci..

[46] Colquhoun H.L., Carroll K., Eva K.W., Grimshaw J.M., Ivers N., Michie S. (2017). Advancing the literature on designing audit and feedback interventions: identifying theory-informed hypotheses. Implement. Sci..

[47] Ivers N., Jamtvedt G., Flottorp S., Young J.M., Odgaard‐Jensen J., French S.D. (2012). Audit and feedback: effects on professional practice and healthcare outcomes. Cochrane Database Syst. Rev..

[48] Shelton R.C., Cooper B.R., Stirman S.W. (2018). The sustainability of evidence-based interventions and practices in public health and health care. Annu. Rev. Publ. Health.

[49] Palinkas L.A., Rhoades Cooper B., Brownson R.C., Colditz G.A., Proctor E.K. (2017). Mixed methods evaluation in dissemination and implementation science. Dissemination and Implementation Research in Health.

[50] Harris P.A., Taylor R., Thielke R., Payne J., Gonzalez N., Conde J.G. (2009). Research electronic data capture (REDCap)—a metadata-driven methodology and workflow process for providing translational research informatics support. J. Biomed. Inf..

[51] Smith J.D., Rafferty M.R., Heinemann A.W., Meachum M.K., Villamar J.A., Lieber R.L. (2020). Evaluation of the factor structure of implementation research measures adapted for a novel context and multiple professional roles. BMC Health Serv. Res..

[52] Armenakis A.A., Bernerth J.B., Pitts J.P., Walker H.J. (2007). Organizational change recipients' Beliefs scale:development of an assessment instrument. J. Appl. Behav. Sci..

[53] Aarons G.A., Ehrhart M.G., Farahnak L.R. (2014). The Implementation Leadership Scale (ILS): development of a brief measure of unit level implementation leadership. Implement. Sci..

[54] Upton D., Upton P. (2006). Development of an evidence-based practice questionnaire for nurses. J. Adv. Nurs..

[55] Brown C.H., Have T.R.T., Jo B., Dagne G., Wyman P.A., Muthén B. (2009). Adaptive designs for randomized trials in public health. Annu. Rev. Publ. Health.

[56] Feldt T., Rantanen J., Hyvönen K., Mäkikangas A., Huhtala M., Pihlajasaari P. (2014). The 9-item Bergen Burnout Inventory: factorial validity across organizations and measurements of longitudinal data. Ind. Health.

[57] May C., Rapley T., Mair F.S., Treweek S., Murray E., Ballini L., Macfarlane A., Girling M., Finch T.L. (2015). Normalization process theory on-line users' manual, toolkit and NoMAD instrument. http://www.normalizationprocess.org.

[58] Luke D.A., Malone S., Prewitt K., Hackett R., Lin J. (2018). The clinical sustainability assessment tool (CSAT): assessing sustainability in clinical medicine settings. Conference on the Science of Dissemination and Implementation in Health; Washington, D.C.

[59] Malone S., McKay V., Prewitt K., Smith J.D., Agulnik A., Luke D. (2020). Validating and enhancing the clinical sustainability assessment tool: a quick assessment for researchers and practitioners. 13th Annual Conference on the Science of Dissemination and Implementation; Washington, DC.

[60] Luke D.A., Calhoun A., Robichaux C.B., Elliott M.B., Moreland-Russell S. (2014). The Program Sustainability Assessment Tool: a new instrument for public health programs. Prev. Chronic Dis..

[61] Gale R.C., Wu J., Erhardt T., Bounthavong M., Reardon C.M., Damschroder L.J. (2019). Comparison of rapid vs in-depth qualitative analytic methods from a process evaluation of academic detailing in the Veterans Health Administration. Implement. Sci..

[62] Damschroder L., Aron D., Keith R., Kirsh S., Alexander J., Lowery J. (2009). Fostering implementation of health services research findings into practice: a consolidated framework for advancing implementation science. Implement. Sci..

[63] Hamilton A.B. (2013). Qualitative Methods in Rapid Turn-Around Health Services Research.

[64] Palinkas L.A., Aarons G.A., Horwitz S., Chamberlain P., Hurlburt M., Landsverk J. (2011). Mixed method designs in implementation research. Adm. Pol. Ment. Health.

[65] Aarons G.A., Fettes D.L., Sommerfeld D.H., Palinkas L.A. (2012). Mixed methods for implementation research: application to evidence-based practice implementation and staff turnover in community-based organizations providing child welfare services. Child. Maltreat..

[66] Glasgow R.E., Askew S., Purcell P., Levine E., Warner E.T., Stange K.C. (2013). Use of RE-AIM to address health inequities: application in a low-income community health center-based weight loss and hypertension self-management program. Translational Behavioral Medicine.

[67] Saldana L., Schaper H., Campbell M., Chapman J. (2015). Standardized measurement of implementation: the universal SIC. Implement. Sci..

[68] Shelton R.C., Chambers D.A., Glasgow R.E. (2020). An extension of RE-AIM to enhance sustainability: addressing dynamic context and promoting health equity over time. Frontiers in Public Health.

[69] Smith J.D., Hasan M. (2020). Quantitative approaches for the evaluation of implementation research studies. Psychiatr. Res..

[70] Stiles P.G., Boothroyd R.A., Snyder K., Zong X. (2002). Service penetration by persons with severe mental illness: how should it be measured?. J. Behav. Health Serv. Res..

[71] Muthén B.O., Muthén L.K. (2000). Integrating person‐centered and variable‐centered analyses: growth mixture modeling with latent trajectory classes. Alcohol Clin. Exp. Res..

[72] Saldana L., Bennett I., Powers D., Vredevoogd M., Grover T., Schaper H. (2020). Scaling implementation of collaborative care for depression: adaptation of the stages of implementation completion (SIC). Adm. Pol. Ment. Health.

[73] Graham A.K., Minc A., Staab E., Beiser D.G., Gibbons R.D., Laiteerapong N. (2019). Validation of the computerized adaptive test for mental health in primary care. Ann. Fam. Med..

[74] Arroll B., Goodyear-Smith F., Crengle S., Gunn J., Kerse N., Fishman T. (2010). Validation of PHQ-2 and PHQ-9 to screen for major depression in the primary care population. Ann. Fam. Med..

[75] Chew-Graham C.A., Lovell K., Roberts C., Baldwin R., Morley M., Burns A. (2007). A randomised controlled trial to test the feasibility of a collaborative care model for the management of depression in older people. Br. J. Gen. Pract..

[76] Unützer J., Katon W., Callahan C.M., Williams J., John W., Hunkeler E., Harpole L. (2002). Collaborative care management of late-life depression in the primary care SettingA randomized controlled trial. J. Am. Med. Assoc..

[77] Katon W.J., Lin E.H.B., Von Korff M., Ciechanowski P., Ludman E.J., Young B. (2010). Collaborative care for patients with depression and chronic illnesses. N. Engl. J. Med..

[78] Ettman C.K., Abdalla S.M., Cohen G.H., Sampson L., Vivier P.M., Galea S. (2020). Prevalence of depression symptoms in US adults before and during the COVID-19 pandemic. JAMA Network Open.

[79] Raghavan R., Brownson R., Colditz G., Proctor E. (2017). The role of economic evaluation in dissemination and implementation research. Dissemination and Implementation Research in Health: Translating Science to Practice.

[80] Jacobson N.S., Truax P. (1991). Clinical significance: a statistical approach to defining meaningful change in psychotherapy research. J. Consult. Clin. Psychol..

[81] Akincigil Ayse, Elizabeth B., Matthews M.S.W. (2017). National rates and patterns of depression screening in primary care: results from 2012 and 2013. Psychiatr. Serv..

[82] Craven M.A., Bland R. (2013). Depression in primary care: current and future challenges. Can. J. Psychiatr..

[83] Overbeck G., Davidsen A.S., Kousgaard M.B. (2016). Enablers and barriers to implementing collaborative care for anxiety and depression: a systematic qualitative review. Implement. Sci..

[84] Wood E., Ohlsen S., Ricketts T. (2017). What are the barriers and facilitators to implementing Collaborative Care for depression? A systematic review. J. Affect. Disord..

[85] Sullivan G., Craske M.G., Sherbourne C., Edlund M.J., Rose R.D., Golinelli D. (2007). Design of the Coordinated Anxiety Learning and Management (CALM) study: innovations in collaborative care for anxiety disorders. Gen. Hosp. Psychiatr..

[86] Mark S., Bauer M.D., Linda McBride M.S.N., William O. (2006). Williford PD, Henry Glick PD, Bruce Kinosian MD, Lori Altshuler MD, et al. Collaborative Care for Bipolar Disorder: Part I. Intervention and Implementation in a Randomized Effectiveness Trial. Psychiatr. Serv..

[87] LaBelle C.T., Han S.C., Bergeron A., Samet J.H. (2016). Office-based opioid treatment with buprenorphine (OBOT-B): statewide implementation of the Massachusetts collaborative care model in community health centers. J. Subst. Abuse Treat..

[88] Engel C.C., Oxman T., Yamamoto C., Gould D., Barry S., Stewart P. (2008). RESPECT-mil: feasibility of a systems-level collaborative care approach to depression and post-traumatic stress disorder in military primary care. Mil. Med..

[89] Craske M.G., Roy-Byrne P., Stein M.B., Donald-Sherbourne C., Bystritsky A., Katon W. (2002). Treating panic disorder in primary care: a collaborative care intervention. Gen. Hosp. Psychiatr..

[90] Mohr D.C., Schueller S.M., Riley W.T., Brown C.H., Cuijpers P., Duan N. (2015). Trials of intervention principles: evaluation methods for evolving behavioral intervention technologies. J. Med. Internet Res..

[91] Smith J.D. (2012). Single-case experimental designs: a systematic review of published research and current standards. Psychol. Methods.

