# Supplementary material for: Collaborative care for depression management in primary care: A randomized roll-out trial using a type 2 hybrid effectiveness-implementation design
Source: Contemp Clin Trials Commun. 2021 Jul 26;23:100823. doi: 10.1016/j.conctc.2021.100823 (PMC8350002; doi:10.1016/j.conctc.2021.100823)

**Additional File 1**

Survey Questions Developed for this Study

**Baseline Pre-Implementation Survey**

AAF-CoCM


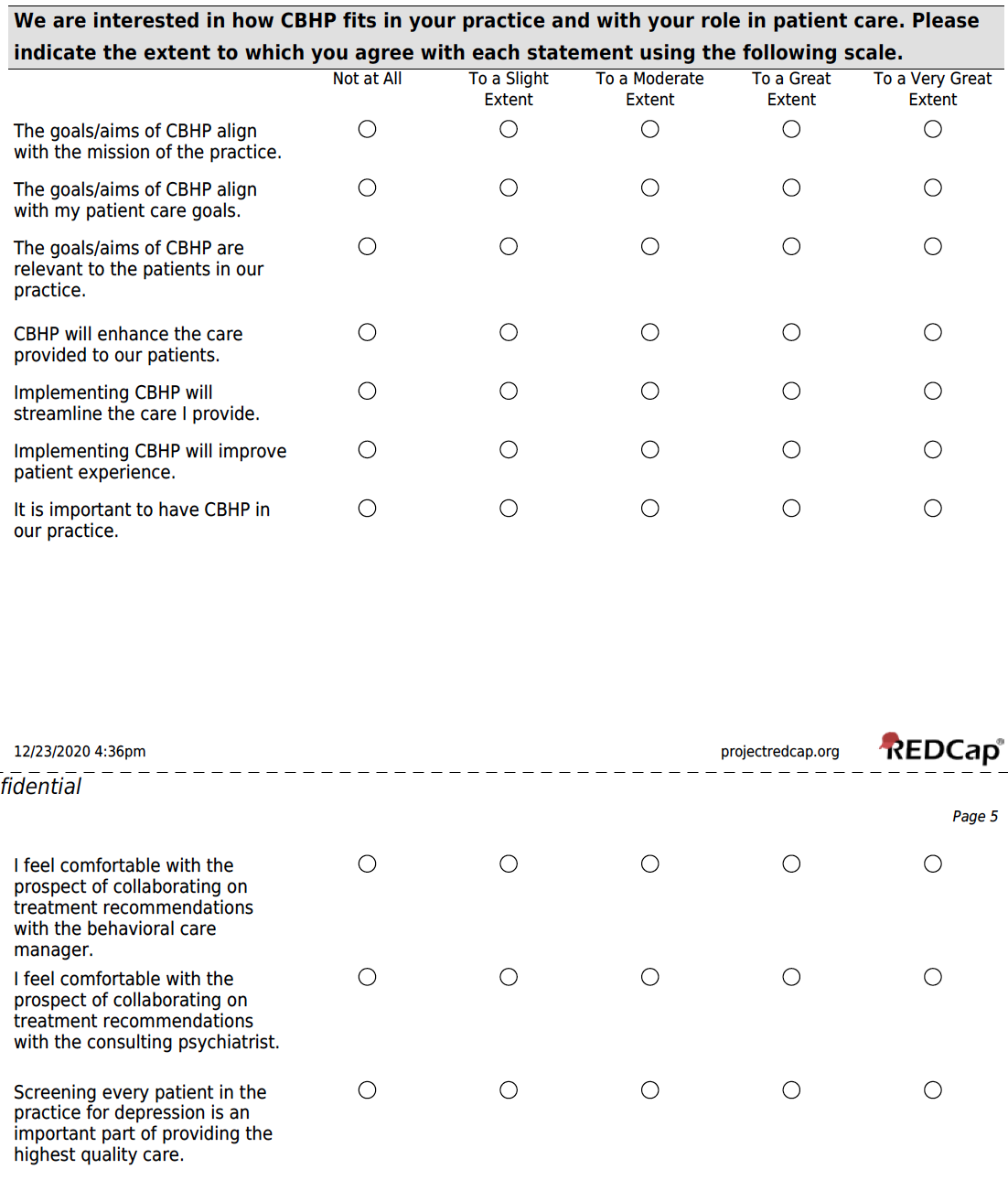


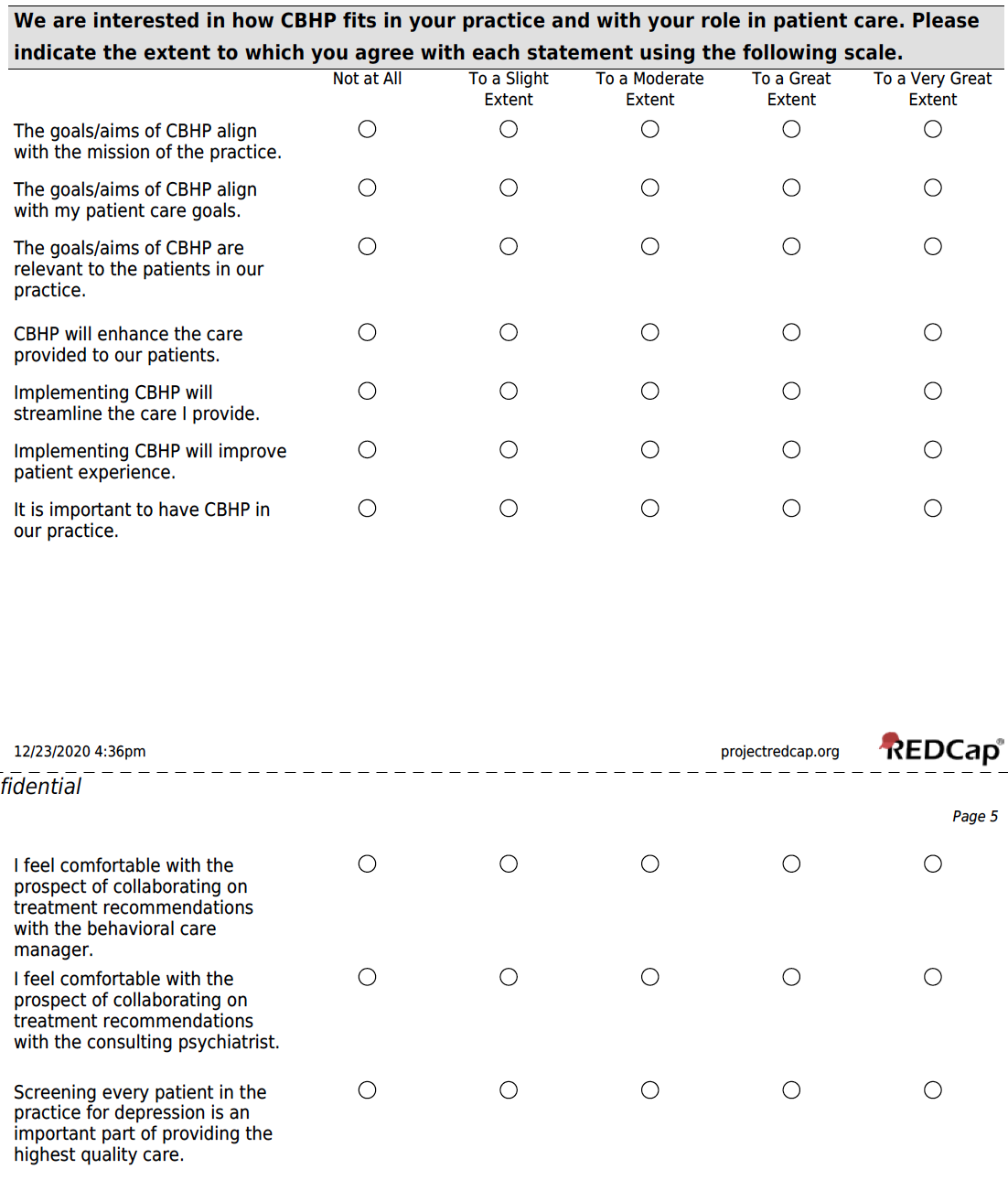


**6-Month Follow-Up Survey**

AAF-CoCM


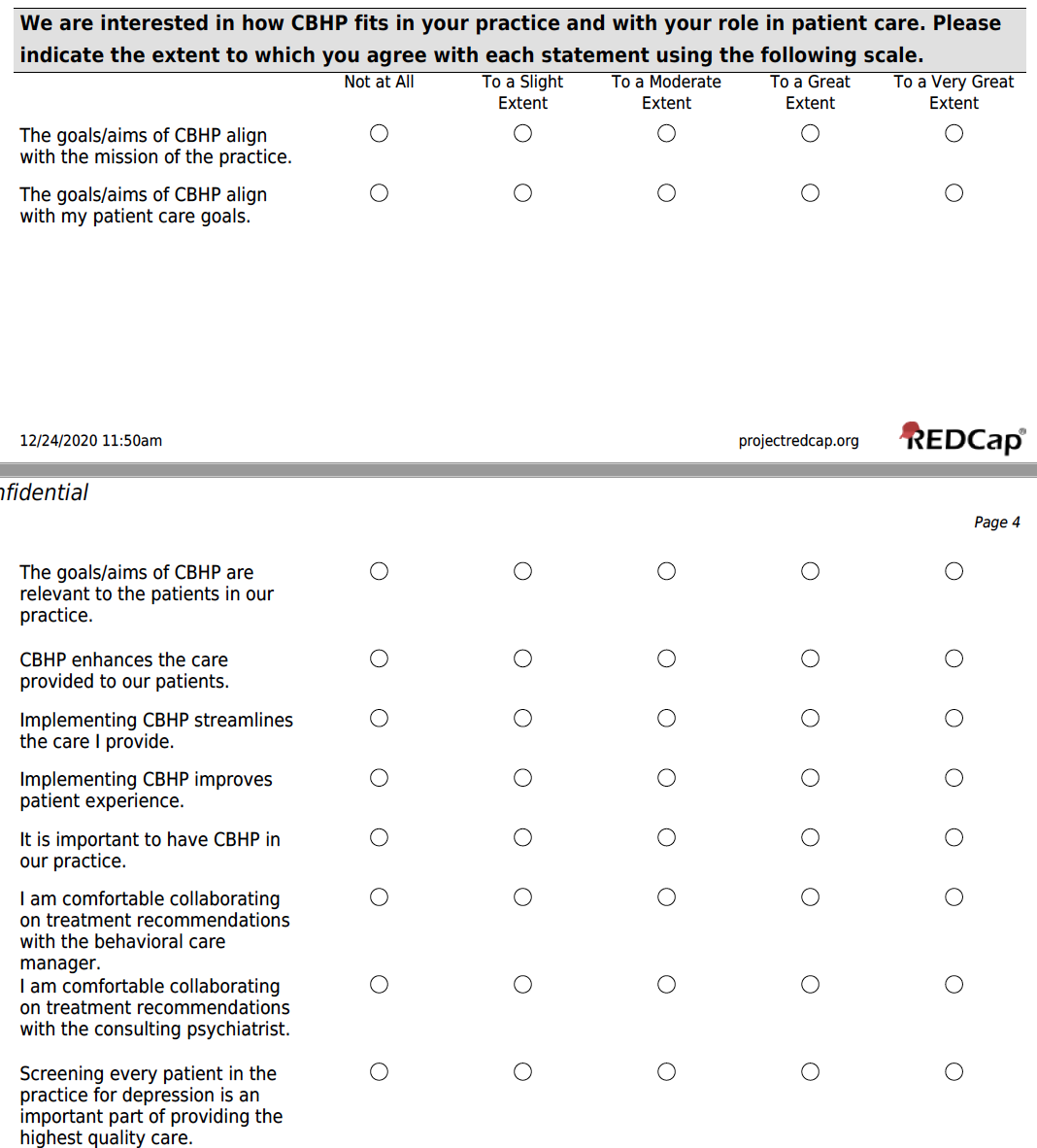


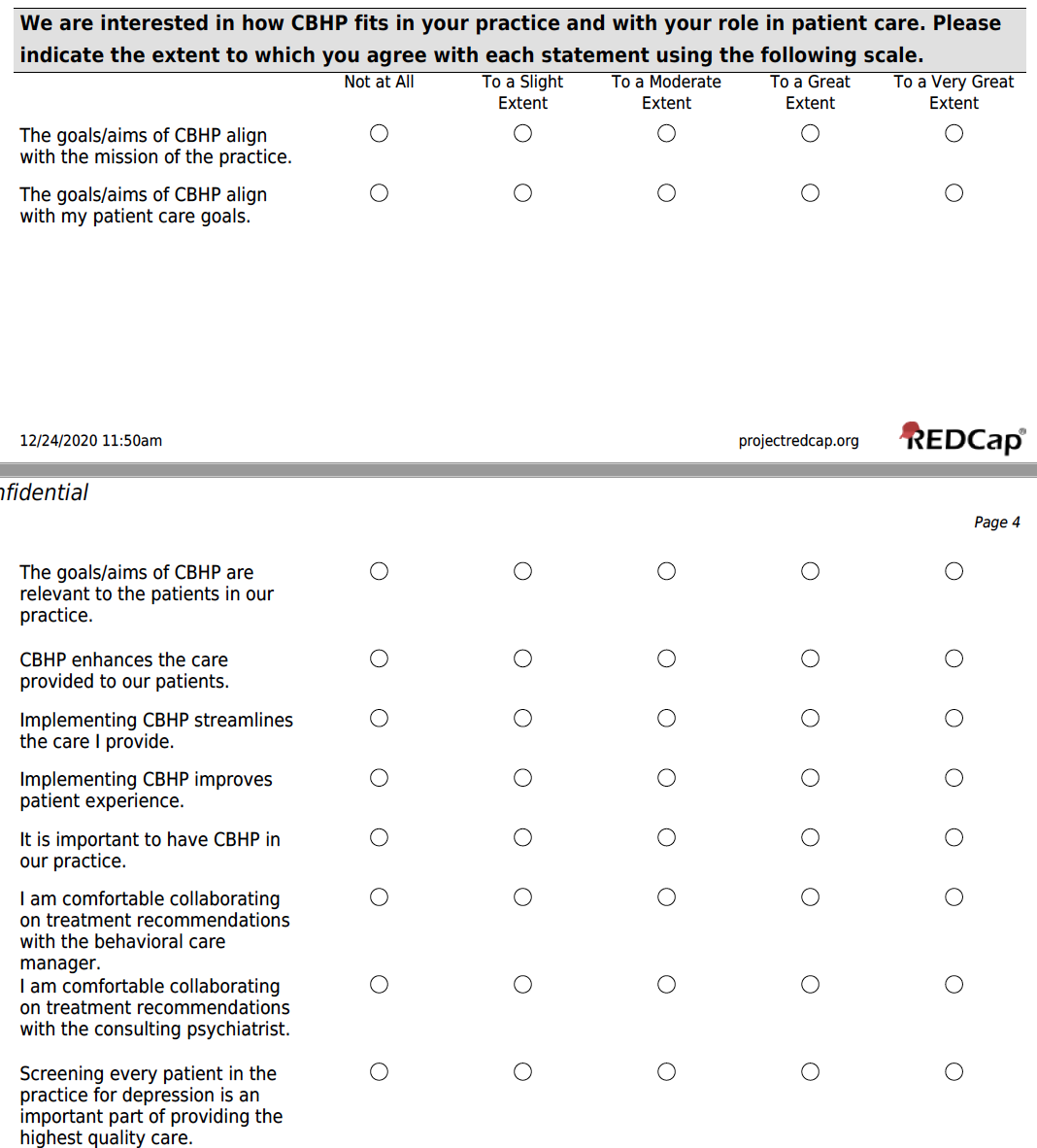


Support


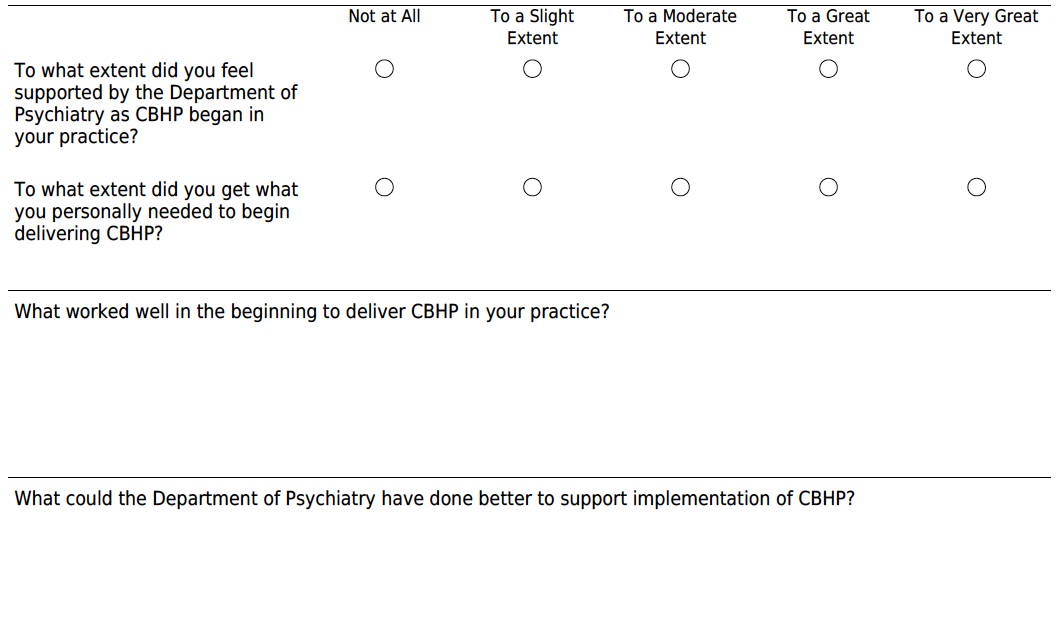


Open Ended


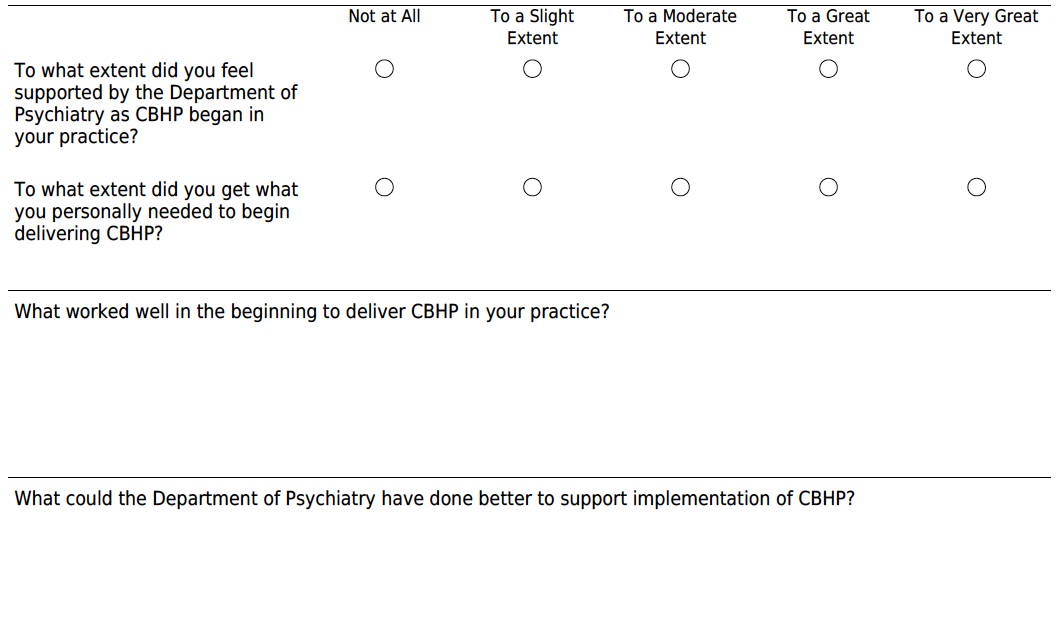


**12-Month Follow-Up Survey**

AAF-CoCM


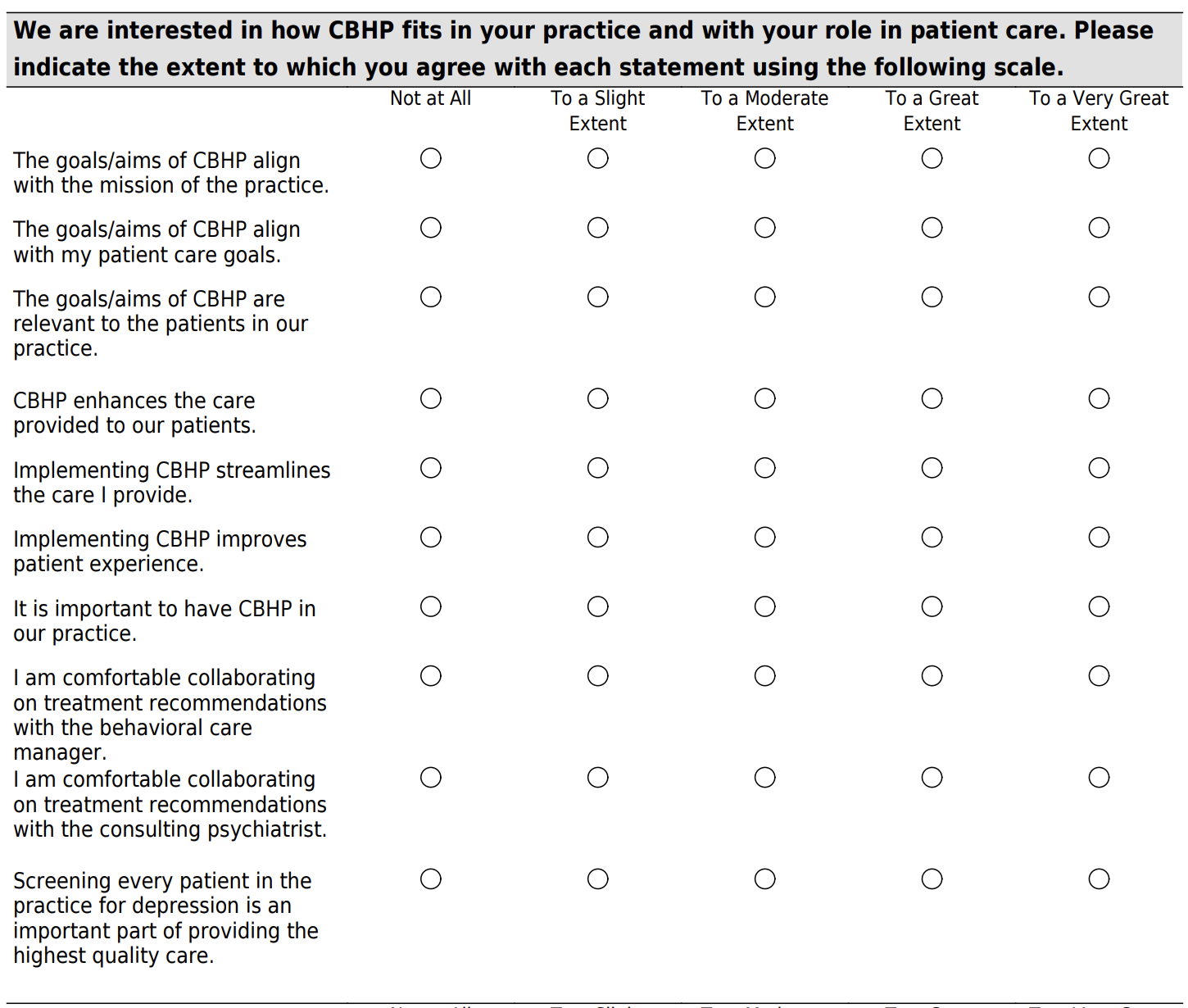


Support


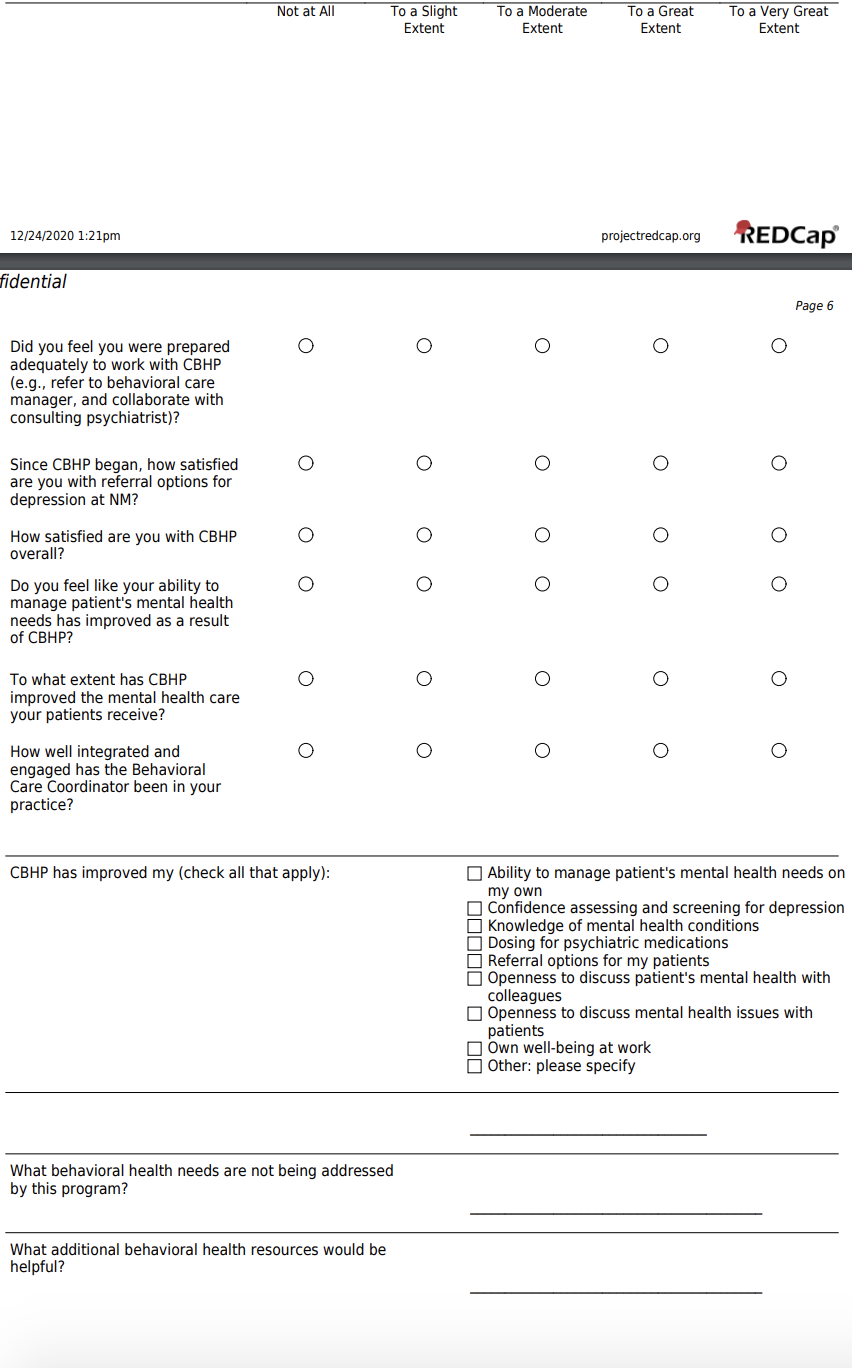


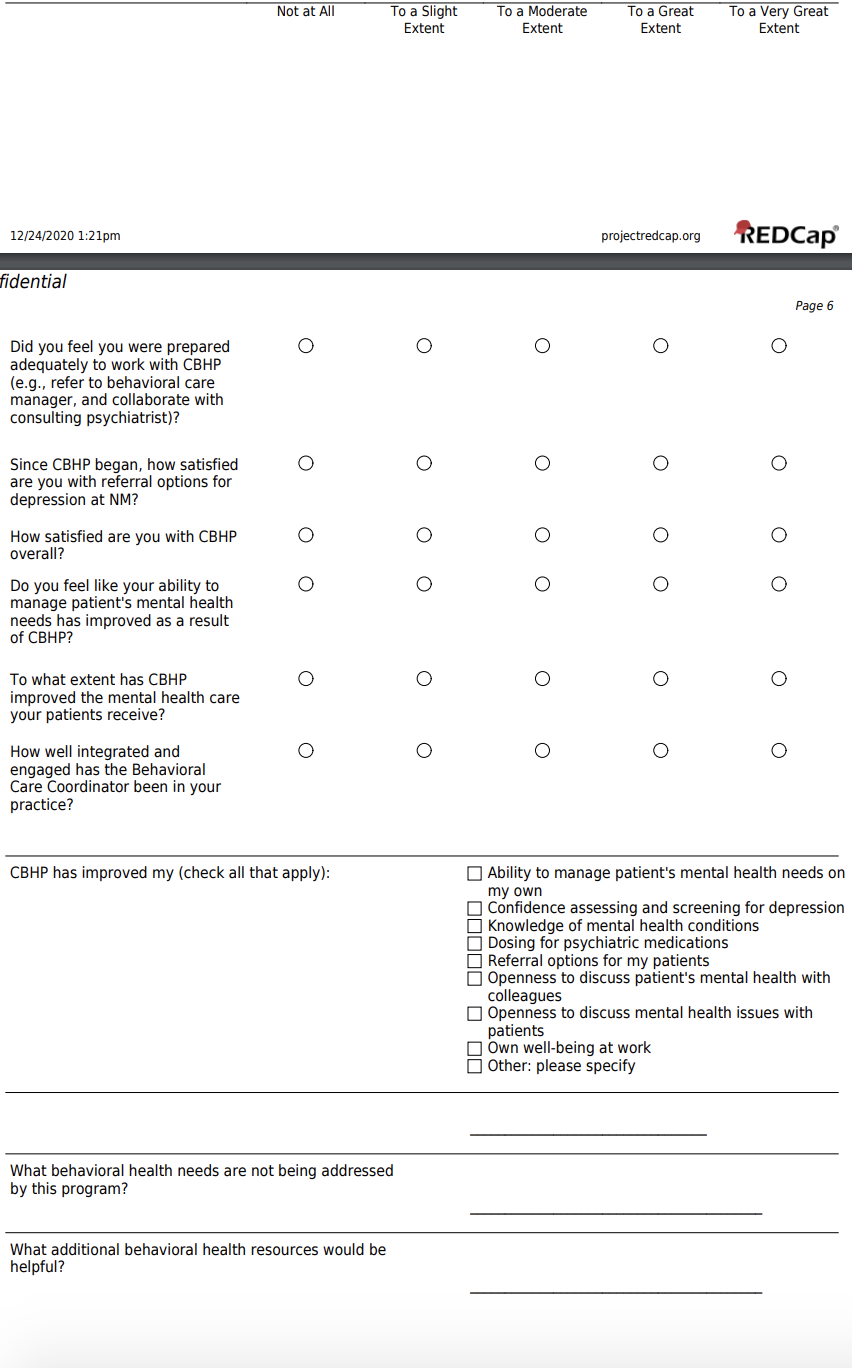


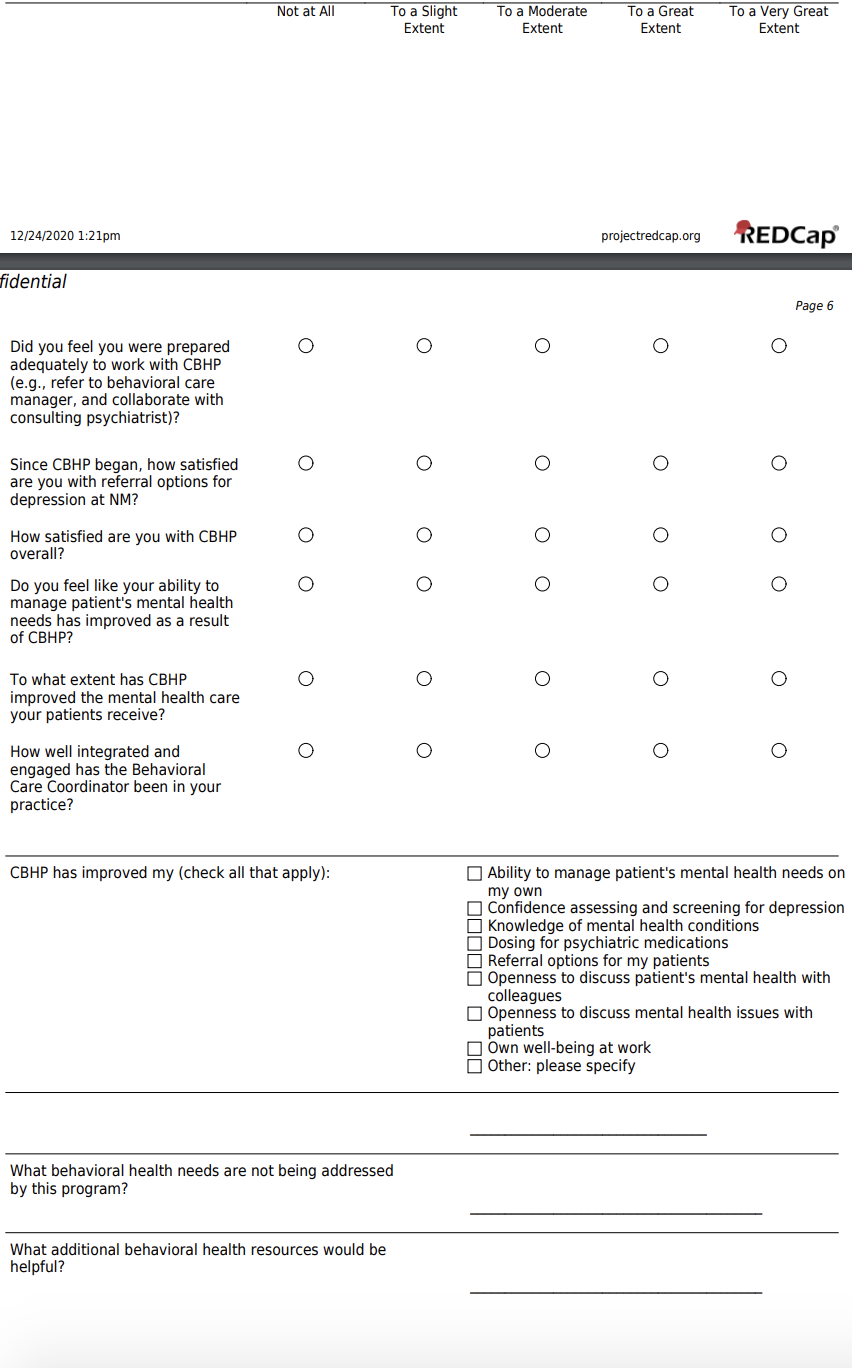


Open-Ended


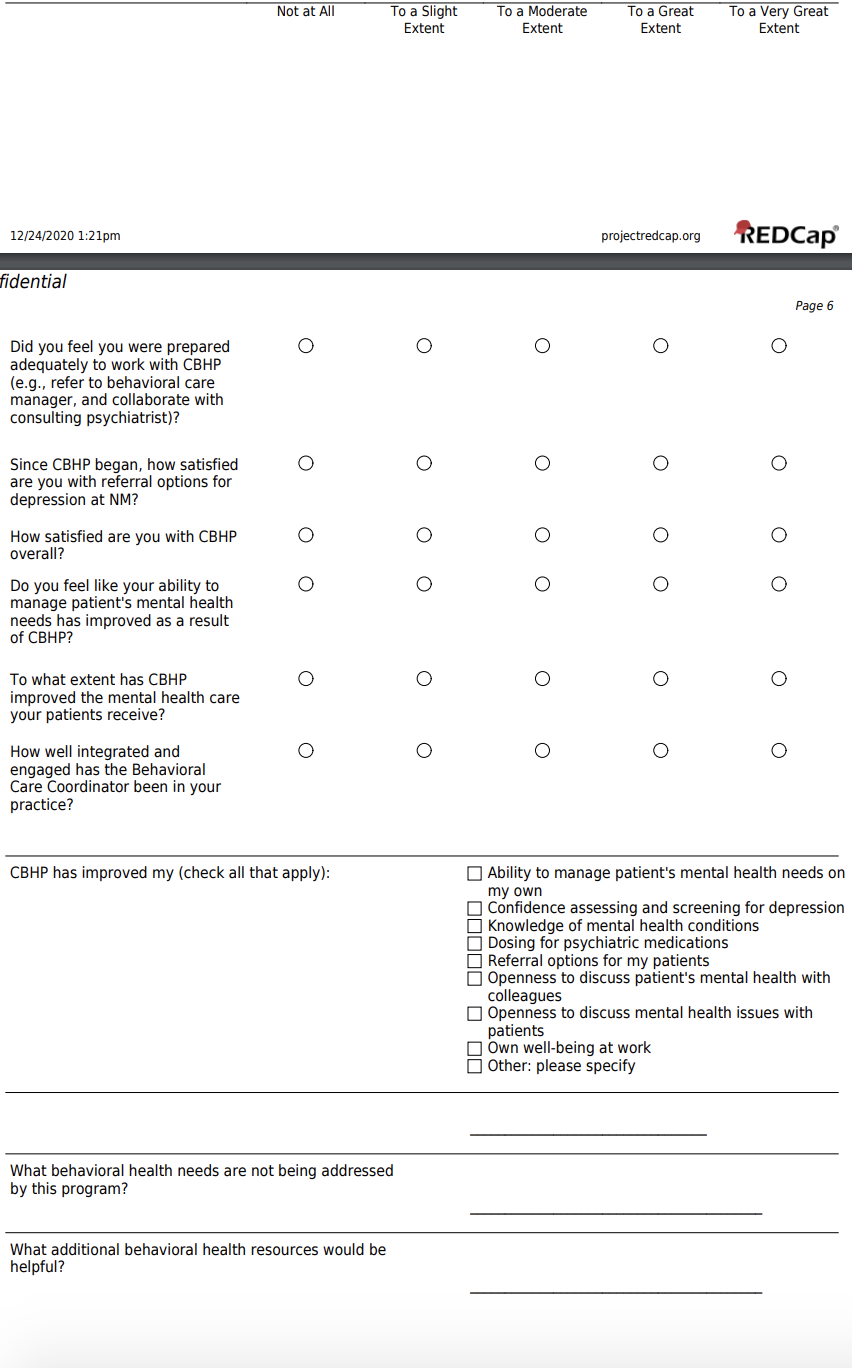


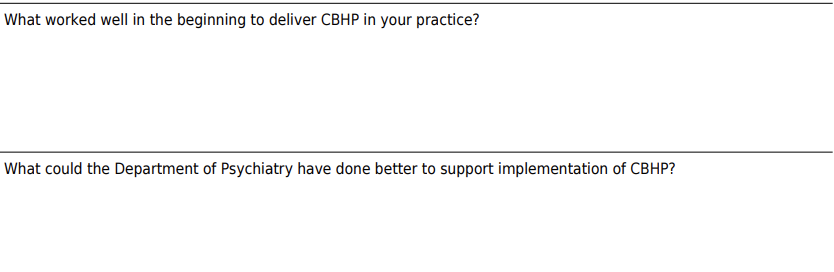


**24-Month Follow-Up Survey**

Support


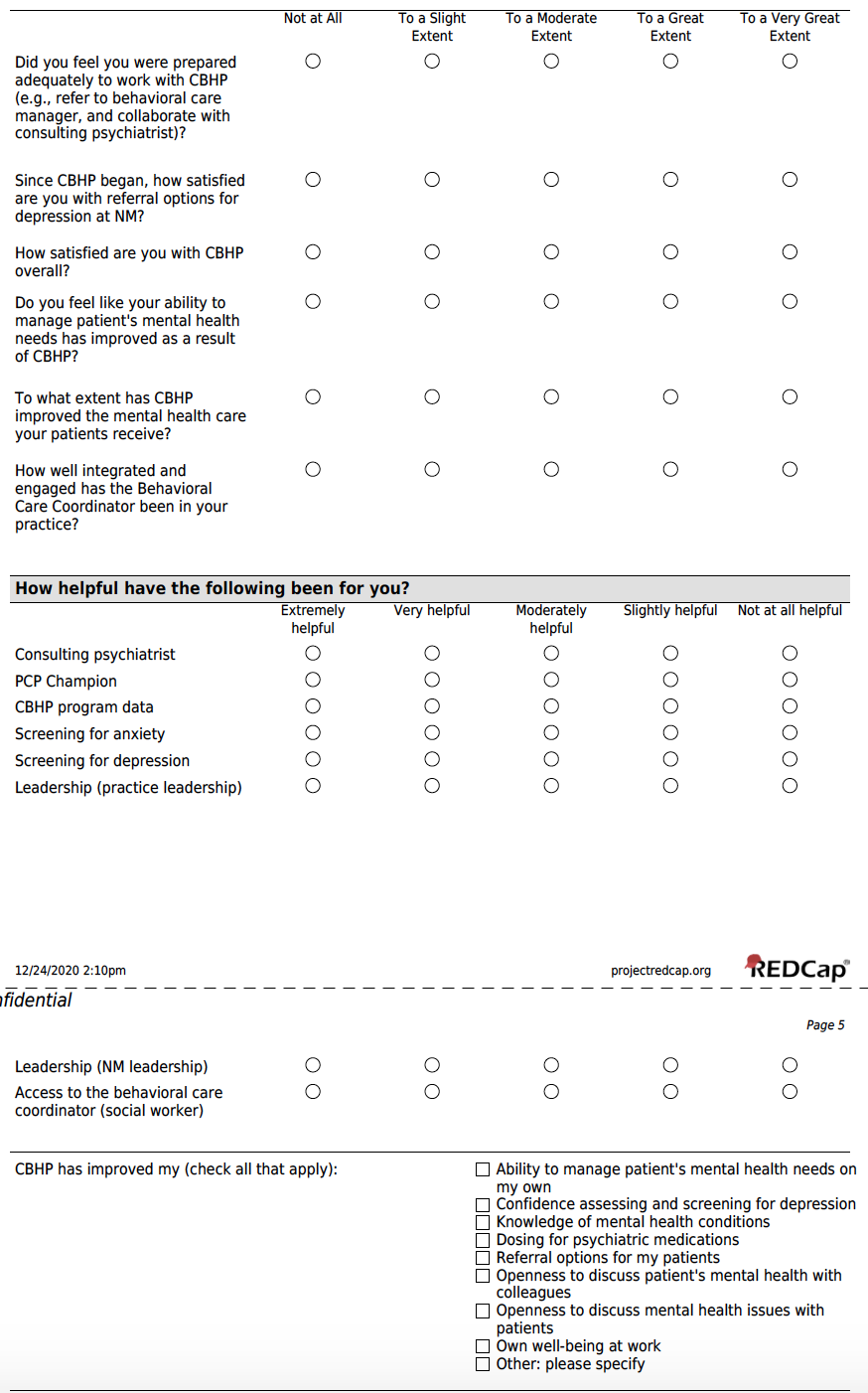


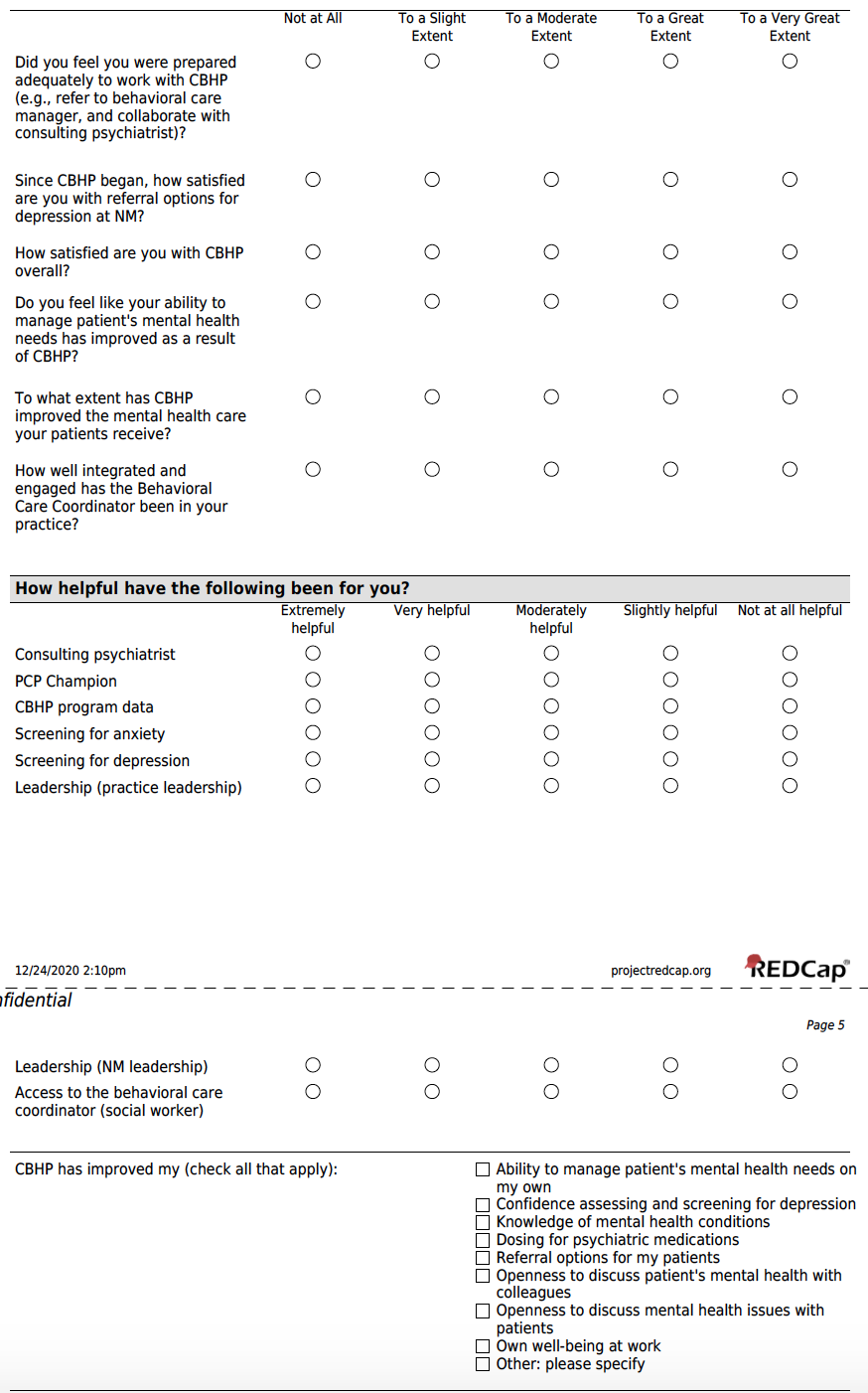


Open-Ended


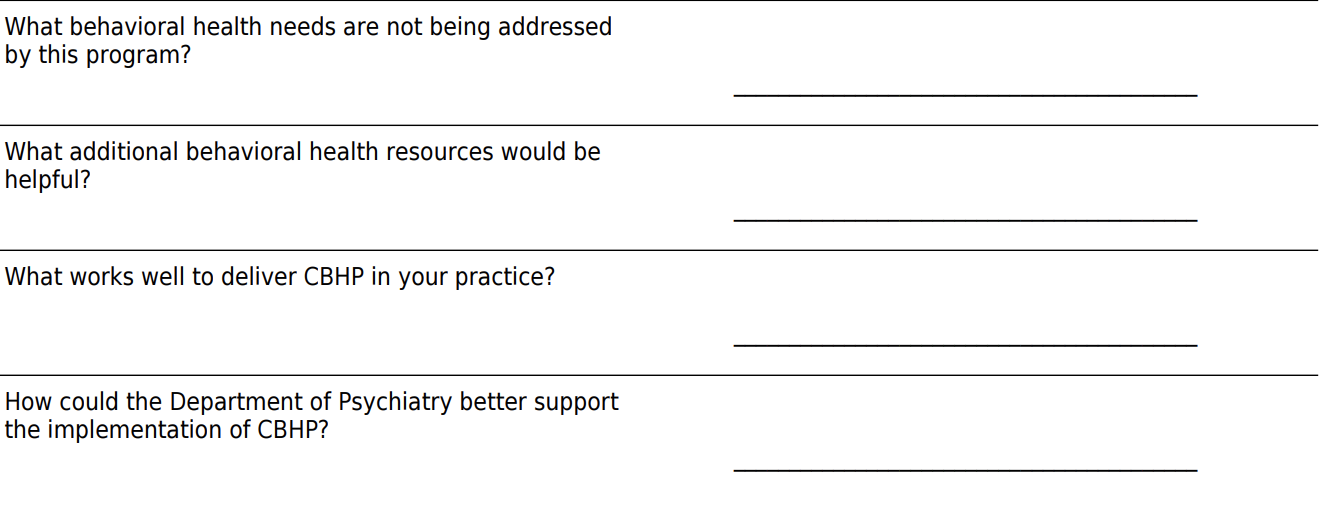

Supplement: Multimedia component 1 [file mmc1.docx]
